# Mannosylation of Virus-Like Particles Enhances Internalization by Antigen Presenting Cells

**DOI:** 10.1371/journal.pone.0104523

**Published:** 2014-08-14

**Authors:** Farah Al-Barwani, Sarah L. Young, Margaret A. Baird, David S. Larsen, Vernon K. Ward

**Affiliations:** 1 Department of Microbiology and Immunology, Otago School of Medical Science, University of Otago, Dunedin, New Zealand; 2 Department of Pathology, Dunedin School of Medicine, University of Otago, Dunedin, New Zealand; 3 Department of Chemistry, Division of Sciences, University of Otago, Dunedin, New Zealand; German Primate Center, Germany

## Abstract

Internalization of peptides by antigen presenting cells is crucial for the initiation of the adaptive immune response. Mannosylation has been demonstrated to enhance antigen uptake through mannose receptors, leading to improved immune responses. In this study we test the effect of surface mannosylation of protein-based virus-like particles (VLP) derived from Rabbit hemorrhagic disease virus (RHDV) on uptake by murine and human antigen presenting cells. A monomannoside and a novel dimannoside were synthesized and successfully conjugated to RHDV VLP capsid protein, providing approximately 270 mannose groups on the surface of each virus particle. VLP conjugated to the mannoside or dimannoside exhibited significantly enhanced binding and internalization by murine dendritic cells, macrophages and B cells as well as human dendritic cells and macrophages. This uptake was inhibited by the inclusion of mannan as a specific inhibitor of mannose specific uptake, demonstrating that mannosylation of VLP targets mannose receptor-based uptake. Consistent with mannose receptor-based uptake, partial retargeting of the intracellular processing of RHDV VLP was observed, confirming that mannosylation of VLP provides both enhanced uptake and modified processing of associated antigens.

## Introduction

The capsid proteins of numerous viruses can spontaneously assemble into highly uniform virus-like particles (VLP) that are morphologically identical to the native virus but do not contain viral genomic material [1]. VLP are strongly immunogenic and can generate long-lasting immunologic memory, consequently their use in particulate subunit vaccines has been studied extensively [2], [3]. In addition to their ability to provide protection against the parent virus, VLPs can also act as effective platforms for the delivery of foreign antigens, such as tumor-associated antigens, through chemical or genetic modification [4], [5].

Vaccination involves the initiation of the adaptive immune response against specific antigens, leading to protection against establishment of disease. One of the key stages in the generation of an efficacious immune response is the internalization of antigenic peptides by antigen presenting cells (APCs). APCs process and present the peptides on major histocompatibility complex (MHC) molecules to naïve T lymphocytes. With the help of co-stimulatory molecules, this can lead to the activation of these cells resulting in either cell-mediated or humoral immunity. Therefore, the augmentation of vaccine internalization by APCs has the potential to greatly enhance vaccine efficacy.

The VLP used in this study is derived from the Rabbit hemorrhagic disease virus (RHDV) and is composed of 180 copies of the viral capsid protein VP60 [6]. RHDV VLP can be readily altered to express model tumor-associated antigens. These include the lymphocytic choriomeningitis virus gp33 and HPV 16 E6 MHC class I epitopes (gp33_33–41_ and E6_48–57_), as well as the ovalbumin MHC class I and II peptides (OVA_257–264_ and OVA_323–339_). Vaccination with RHDV VLP expressing these model tumor associated antigens leads to a delay in tumor development *in vivo* using appropriate murine tumor models [4], [7]–[9].

RHDV VLP are internalized by APCs primarily through phagocytosis and macropinocytosis [10]. In addition to these general mechanisms of uptake, APCs can internalize antigens through receptor-mediated endocytosis. The scavenger receptor, Fc receptors and C-type lectins (dendritic cell-specific ICAM-3-grabbing nonintegrin (DC-SIGN) and CD206) are examples of surface receptors that allow more targeted internalization of antigens by APCs [11], [12]. C-type lectins such as CD206 are highly expressed on antigen presenting cells such as dendritic cells (DCs) and macrophages [13]. Numerous studies have demonstrated that targeting mannose receptor-based internalization can lead to an enhancement in antigen presentation on MHC class I and II, leading to an augmentation of both cell-mediated and humoral immunity [14]–[20]. These two arms of the immune response have been shown to work together synergistically to resolve disease and generate effective immunological memory.

Another key determinant of vaccine efficacy is antigen processing within APCs. For the induction of robust anti-tumor immunity, antigens must be processed and presented to CD8^+^ T cells on MHC I molecules. As most vaccines are delivered extracellularly, the processing and presentation of antigens on MHC I molecules occurs though a process termed cross-presentation. RHDV VLP associated epitopes are cross-presented by DCs though the receptor-recycling pathway in which antigen containing early endosomes fuse with lysosomes, where the antigens are processed, bound to MHC-I molecules then recycled to the cell membrane [4], [10]. This is one of many pathways by which antigens can be cross-presented by APCs. Internalization of antigens through the mannose receptor has been demonstrated to target the endosome-to-cytosol pathway of cross-presentation, in which antigens internalized into early endosomes are transferred to the cytoplasm where they undergo proteasomal degradation and further processing though the endoplasmic reticulum [14], [18]. Therefore, mannosylation of RHDV VLP could potentially provide an alternate route of processing within APCs.

Our work explores the potential of enhancing RHDV VLP functionality by targeting mannose-specific internalization. A new pathway for the synthesis of a monomannoside and a novel dimannoside was developed, to allow conjugation of mannosides to VLP while retaining VLP stability. Furthermore, fluorescently labeled VLP conjugated to the mannoside or dimannoside were used to determine the effect of VLP mannosylation on VLP internalization and processing by key APCs.

## Experimental Procedures

### General Methods for Mannoside Synthesis

Solvents were purchased from commercial sources and used without further purification, except dichloromethane, tetrahydrofuran and toluene that were dried using the PURE SOLV MD-6 solvent purification system. NMR spectra were recorded on 500 MHz AR Premium Shielded Spectrometer. Chemical shifts are reported in δ (ppm) using residual solvent signals from deuterated solvents as references; coupling constants are reported in Hertz [Hz]. Assignments were made with the aid of COSY, HSQC, and HMBC experiments. Infrared (IR) spectra were recorded with a Burker Optics Alpha FT-IR spectrophotometer with a diamond Attenuated Total Reflectance top plate. High-resolution mass spectra (HR-MS) were recorded on a Bruker microTOFQ mass spectrometer using an electrospray ionisation (ESI) source in the positive mode. Specific rotation ([α]_D_^T^) values were recorded on a Jasco DIP-1000 digital polarimeter using a 100 mm cell with a 3.5 mm aperture, and the rotation measured at 589 nm (sodium D line) at ambient temperature T (°C). Thin layer chromatography was performed on aluminium-backed silica gel 60 (0.20 mm) plates (Merck, Darmstadt, Germany) and compounds detected with 5% w/v dodecaphosphomolybdic acid in ethanol after heating. Column chromatography was performed using silica gel 60 (230–400 mesh). Detailed experimental procedures for mannoside synthesis as well as ^1^H and ^13^C NMR spectra are included in Data S1 and Data S2.

### Generation of RHDV VLP

RHDV VLP and RHDV VLP engineered to express the model tumor antigen SIINFEKL (VLP.SIINr), were expressed using a recombinant *Autographa californica* Nucleopolyhedrovirus (AcMNPV), as described previously by McKee et al [9]. Briefly, to generate VLP.SIINr a DNA sequence encoding the ovalbumin epitope SIINFEKL was added to the N-terminus of the RHDV VP60 gene by PCR extension and used to generate a recombinant baculovirus by homologous recombination. To express VLP, suspension cultures of Sf21 insect cells were infected with recombinant baculovirus at a multiplicity of infection of 1 and incubated at 27°C with shaking at 125 rpm. After 3 days, cells were lysed with 0.5% Triton X-100 and the VLP purified using differential centrifugation, followed by a two step 1.2 and 1.4 g mL^−1^ CsCl gradient (centrifuged at 100,000 g at 27°C for 18 h). The VLP band was harvested and VP60 expression was confirmed by 10% SDS PAGE gel, visualized by Coomassie Brilliant Blue G-250 staining (BD Biosciences, San Jose, CA, USA). To confirm VLP assembly, VLP were negatively stained with phosphotungstic acid and viewed with the Philips CM100 transmission electron microscope at the Otago Centre for Electron Microscopy. VLP concentrations were determined by A280 absorbance by NanoDrop (Thermo Scientific, Rockford, IL, USA), using an ε of 78000 M^−1^ cm^−1^ and a molecular weight of 60 kDa. All VLP modifications and dialysis were performed in phosphate buffered saline (PBS) (0.2 M, pH 7.3) containing 0.3 M NaCl.

### Fluorescent Labeling of VLP

Purified VLP in PBS were coupled to 1∶1 molar equivalent of N-hydroxysuccinimide (NHS)-DyLight 633 (Thermo Scientific) to VP60 for 30 min at room temperature. Following removal of unconjugated DyLight 633 by dialysis, coupling was confirmed by SDS-PAGE (visualized under UV light), and quantified by determining the DyLight:VP60 molar ratio using a NanoDrop (DyLight 633 = λ_max_ 627 nm, ε 170000 M^−1^ cm^−1^).

### Mannosylation of VLP

VLP in PBS was either left without further modification, or conjugated to a 50 molar excess of the mannosides (compounds **7** and **12**), for 3 h at room temperature then overnight at 4°C. After mannosylation, unconjugated mannosides were removed by dialysis and coupling was confirmed by mass-spectrometry. Mannosylation was performed on DyLight labeled VLP, to allow tracking of uptake, and on unlabeled VLP to allow carbohydrate analysis and estimation by lectin blot and Carbohydrate Estimation Kit (Thermo Scientific).

### Mass-spectrometry

To confirm mannoside conjugation and identify conjugation sites, modified VLP were run on a 10% SDS-PAGE gel, the VP60 band excised and submitted to the Otago Centre for Protein Research. Following tryptic digest, the peptides were analyzed using a MALDI-TOF/TOF mass spectrometer to confirm the conjugation of the mannosides to RHDV VLP. The mannosylation sites were then marked on an I-TASSER [21], [22] generated RHDV VP60 model, to identify the location of these sites.

### Lectin Blot

To confirm the glycosylation of RHDV VLP, the protein was run on a 10% SDS-PAGE gel and either stained with Coomassie Blue or transferred onto an Immobilon polyvinylidene fluoride (PVDF) membrane (Millipore, Carlsbad, CA, USA). The PVDF membrane was blocked for an hour with 0.5% gelatin (Roche, Indianapolis, IN, USA) in 20 mM Tris buffered saline pH 7.5 containing 150 mM NaCl, 0.25 mM CaCl_2_ and MnCl_2_ with 0.1% Tween 20 (TBS-T). The membrane was washed in TBS-T and incubated for an hour with 5 µg mL^−1^ FITC conjugated Lectin from *Pisum sativum* (Sigma Aldrich, St. Louis, MO, USA) in TBS-T. After washing, the signal was visualized under UV light using a ChemiDoc system (Bio-Rad, Hercules, CA, CA, USA).

### Carbohydrate Quantification

To estimate the mannose:VP60 ratio on the mono- and di-mannosylated VLP, the Glycoprotein Carbohydrate Estimation Kit was used. The VLP samples (0.04 mM), glycoprotein standards (included in kit) and D-mannose standard (1–0.01 mM) were diluted in PBS, treated with sodium meta-periodate to oxidize the mannose groups to aldehydes, then reacted with the Glycoprotein Detection Reagent (following manufacturer’s procedure). The resultant absorbance was read at 550 nm and the extent of mannose conjugation was determined by the difference in glycosylation between mannosylated VLP and non-mannosylated VLP.

### Animals- Source and Ethics

Specific-pathogen free C57Bl/6 and OTI mice were sourced from the Hercus Taieri Research Unit, University of Otago, Dunedin, New Zealand. All mice were euthanized by cervical dislocation and experiments were conducted in accordance with the ethics granted by the University of Otago Animal Ethics Committee (permit number: AEC10/13). All efforts were made to minimize suffering. Human blood was donated by volunteers with written consent and the approval of the University of Otago Ethics Committee for Human Participants (permit number: H13/122).

### Generation of human monocyte-derived DCs and macrophages from peripheral blood

Peripheral blood mononuclear cells (PBMCs) were isolated from human donors using a Ficoll-Paque separation gradient. Monocytes were labeled with anti-CD14 MicroBeads (Miltenyi Biotech, Auburn, CA, USA) and separated according to the manufacturers directions, using the AutoMACS Pro (Miltenyi Biotech). Monocytes were resuspended at 1×10^6^ cells mL^−1^ in RPMI (Gibco, Green Island, NY, USA) containing 10% FCS and penicillin/streptomycin (100 U mL^−1^ Penicillin and 100 µg mL^−1^ streptomycin). Monocytes were cultured for seven days in media supplemented with 2-mercaptoethanol (55 µM) and 50 ng ml^−1^ recombinant human granulocyte-macrophage colony-stimulating factor (rhGM-CSF) to allow differentiation into macrophages. DCs were generated by culturing monocytes for six days in media supplemented with 25 ng mL^−1^ rhGM-CSF and 25 ng mL^−1^ recombinant human interleukin 4. Cells were cultured at 37°C in the presence of 5% CO_2_.

### Immunofluorescent Analysis of Mannosylated RHDV VLP Internalization

Splenocytes from C57Bl/6 mice were treated with ammonium chloride to lyse the red blood cells. Remaining white blood cells were resuspended at 1×10^6^ cells/mL in complete Iscove's Modified Dulbecco's Medium (cIMDM), IMDM+GlutaMAX (Gibco-Invitrogen) containing 2-mercaptoethanol (55 µM), penicillin/streptomycin (100 U mL^−1^ penicillin and 100 µg mL^−1^ streptomycin) and 5% heat-inactivated fetal calf serum (FCS).

Murine splenocytes and human monocyte-derived DCs and macrophages (1×10^6^) were pulsed with 0.125 µM of DyLight labeled VLP, monomannose-VLP or dimannose-VLP and incubated at 37°C or 4°C. After incubation for 1 h, 4 h or 24 h, cells were stained with Fixable Live Dead Yellow or Fixable Live Dead Violet (Invitrogen, Eugene, OR, USA) to allow selective analysis of live cells, then treated with Fc block (BD Biosciences), to block non-specific binding of antibodies to Fc receptors. To identify different cell types, cells were stained with antibodies (BioLegend, San Diego, CA, USA) against the following epitopes; murine DCs (PE-CD11c, clone N418), murine macrophages (PE-F4/80, clone BM8), murine B cells (FITC-B220, clone RA3-6B2), murine T cells (PerCP/Cy5.5-CD3, clone 17A2), human DCs (PE-CD11c, clone 3.9) and human macrophages (PE-CD64, clone 10.1). To confirm the role of the mannosides in the enhanced uptake, cells were pulsed with mannan (Sigma Aldrich) and incubated for 15 min before the addition of the different VLP. Following a titration of mannan (Figure S1) 3 mg mL^−1^ was used to inhibit mannose specific internalization.

Fluorescence was measured using a Gallios flow cytometer (Beckman Coulter, Franklin Lakes, NJ, USA) and analyzed using FlowJo software version 8.8.6 (TreeStar Inc., Ashland, OR, USA). Statistical analysis of the results was performed using GraphPad Prism version 6.0b. (GraphPad Software, La Jolla, CA, USA) Significance was determined using two-way ANOVA with a Bonferroni post-hoc test.

### Analysis of VLP processing by murine bone marrow derived DCs (BMDCs)

Bone marrow was isolated from the hind legs of C57BL/6 mice. Red blood cells were lysed with ammonium chloride. The remaining white blood cells were cultured for six days in cIMDM containing 20 ng mL^−1^ mouse granulocyte-macrophage colony stimulating factor (mGMCSF) (R&D systems). On day six BMDCs (5×10^5^ cells mL^−1^) were treated with the inhibitors lactacystin (20 µM) or primaquine (50 µM) for 15 min, washed in PBS, then pulsed with 0.83 µM mL^−1^ of VLP.SIINr, monomannose-VLP.SIINr, dimannose-VLP.SIINr, ovalbumin or SIINFEKL peptide and incubated at 37°C for 24 h. OTI T cells were added at a DC:T-cell ratio of 1∶10. After 72 h, IFN-γ levels were measured by enzyme-linked immunosorbent assay.

## Results and Discussion

### Synthesis of Mannosides

There are numerous C-type lectins found on the surface of APCs, which bind to a variety of carbohydrates. Two of the most well characterized lectins with a high affinity for mannosylated ligands are DC-SIGN and CD206. Human DC-SIGN has a higher affinity for internal mannosides of complex oligosaccharides structures, while CD206 binds to terminal mannose residues [23], [24]. CD206 displays higher affinity for branched trimannosides than monomannosides [25], [26], however Espuelas *et. al.* found that dimannosylation of liposomes was just as effective as tetra-mannosylation at enhancing liposome uptake [27]. Moreover, α-linked carbohydrates are preferred by human CD206 over β-linked carbohydrates [25]. Therefore, to target mannose specific uptake of the VLP, the compounds synthesized were the known monomannoside **7** (N-Succinimidyl 6-[α-D-mannopyranosyloxy]hexanoate) [28] and the novel 1,2-α-linked dimannoside **12** (N-Succinimidyl 6-[2-O-(α-D-mannopyranosyl)-α-D-mannopyranosyloxy] hexanoate). Both these compounds possess a short α-glycosidic linker and a terminal N-succinimido (NHS) ester, to allow conjugation to free primary amines on the surface of the VLP, leading to formation of a stable covalent amide bond. NHS was chosen over isothiocyanate, another commonly used amine reactive group, as optimal isothiocyanate conjugation requires a high pH >9. We found that purified RHDV VLP is unstable above pH 8 (data not shown), which is consistent with the findings of Fernendez *et al.* [29]. NHS conjugation has a lower optimal pH (∼8.3) with efficient conjugation at pH 7.3, at which VLP retains stability.

**Figure 1:**
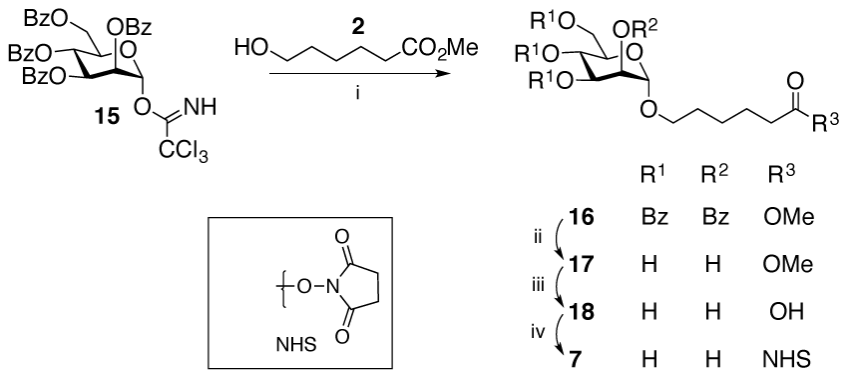
Alternate synthesis of mannose-NHS (7) [28].

**Figure 2:**
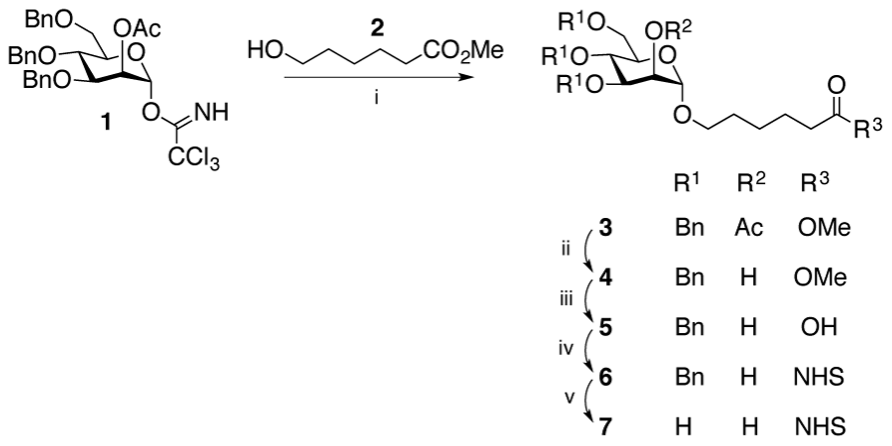
Synthesis of mannose-NHS (7). Conditions: (i) TMSOTf, dichloromethane, 0°C, 1 h, 82%; (ii) NaOMe, MeOH, dichloromethane, RT, 1 h, 70%; (iii) NaOH, THF, reflux, overnight, 90%; (iv) DCC, NHS, THF, RT, overnight, quantitative yield; (v) H_2_, Pd(OH)_2_/C, ethyl acetate, RT, overnight, 77%.

Monomannoside **7** (Figure 1) has been previously reported by Furneaux *et al.* [28] and was prepared from tetra-O-benzyl-D-manopyranosyl trichloroacetimidate **15** and methyl 6-hydroxyhexanoate **2** (Figure 1). Hydrolysis of the methyl ester and benzoate protecting groups of the resulting glycoside followed by activation of the carboxylic acid group with N-hydroxysuccinimide (NHS) gave the monomannoside **7**. Although their approach was successful, it did not provide an intermediate that could be used for the synthesis of dimannoside **12**. Our strategy overcame this issue (Figure 2). Experimental details for the synthesis of monomannose **7** dimannoside 12 and intermediate compounds (**1–6, 8–11**) are detailed in Data S1. The synthesis of monomannoside **7** started with the trimethylsilyl trifluoromethane sulfonate catalyzed glycosylation of **2** [30] and glycosyl trichloroacetimidate **1** [31] which gave glycoside **3** in 82% yield. The use of a glycosyl donor with a participating group at C2 ensured the formation of the α-linked glycoside. This was confirmed from the coupled ^13^C NMR spectrum, where the coupling constant for the anomeric carbon signal (δ = 97.91) was ^1^ *J*_CH_ 169.8 Hz, within the range of an α anomer (∼170–175 Hz) [32]. Sequential hydrolyses of the acetate group at C2 via ester **4**, then of the methyl ester gave acid **5** in a 63% yield over both steps. DCC promoted coupling of NHS to the carboxylic acid produced NHS ester **6**, which was then deprotected by catalytic hydrogenolysis to afford the monomannoside **7** with a 77% yield over the two steps. The spectral data of **7** were consistent with those reported by Furneaux et al. [28].

**Figure 3:**
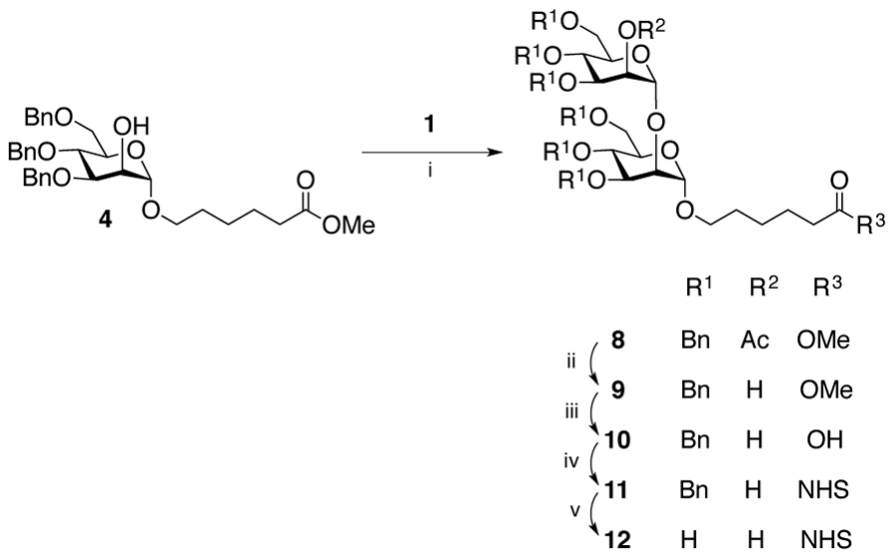
Synthesis of dimannose-NHS (12). Conditions: (i) TMSOTf, dichloromethane, 0°C, 1 h, 89%; (ii) NaOMe, MeOH, dichloromethane, RT, 1 h, 92%; (iii) NaOH, THF, reflux, overnight, 81%; (iv) DCC, NHS, THF, RT, overnight, quantitative yield; (v) H_2_, Pd(OH)_2_/C, THF, RT, overnight, 75%.

Dimannoside **12** (Figure 3) was prepared by glycosylation of intermediate **4** with donor **1** in the same manner to give the α-linked disaccharide **8** (^1^ *J*_CH_ of the anomeric carbons were 169.8 and 170.1 Hz). Sequential hydrolysis of acetate and methyl ester, DCC promoted coupling of NHS, and removal of the benzyl protecting groups gave the target dimannoside **12**, via intermediates (**9**–**11**), in good overall yield (50%).

### Mannosylation of RHDV VLP

To generate RHDV VLP, the RHDV capsid protein VP60 was expressed in a baculovirus expression system and purified by differential and gradient centrifugation. VP60 production and purification were confirmed by SDS-PAGE and mass spectrometry. VLP assembly was confirmed by electron microscopy. This synthesis and processing of RHDV VLP leads to consistent production of purified particles ∼40 nm in diameter, which have been shown to be effective antigen delivery platforms [4], [6], [10], [33].

**Figure 4:**
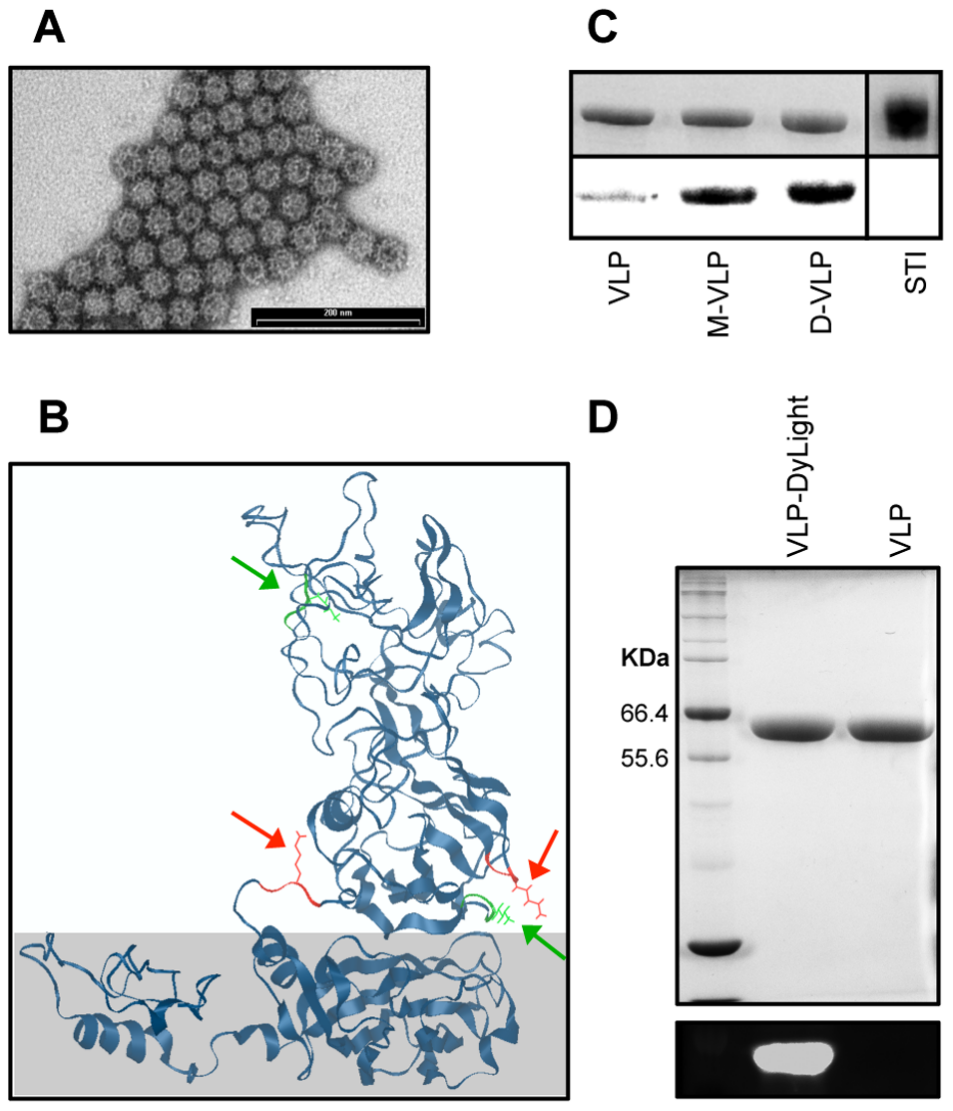
VLP surface modification. (A) TEM micrograph of monomannose-VLP (negatively stained with PTA). Scale bar = 200 nm. (B) RHDV VP60 model generated by I-TASSER, with the VLP P domain (top) and the shell domain (bottom, shaded). Mass spectrometry confirmed mannosylation sites displaying both monomannoside and dimannoside conjugation are depicted in red (lysines 232 and 457), while sites with only monomannoside conjugation are green (lysines 299 and 562). (C) Top: Coomasie blue stained SDS-PAGE gel of VLP, dimannose-VLP (D-VLP), monomannose-VLP (M-VLP) and soybean trypsin inhibitor (STI) as a negative control. Bottom: *Pisum sativum* lectin blot of different VLP. (D) SDS-PAGE analysis of DyLight labeled VLP, stained with Coomassie blue (top) or viewed under UV light (bottom).

Each VLP is composed of 180 copies of VP60 [34], each containing six lysine residues, and thus six potential sites of NHS conjugation. To confirm the ability of the synthesized mannosides to conjugate to these lysine residues, an excess of the monomannoside **7** or dimannoside **12** were conjugated to purified VLP. The resulting mannosylated VLP were found to have retained their structure and shape, as apparent by the 40 nm particles depicted in the TEM micrograph (Figure 4A). Moreover, mass spectrometric analysis of the mannosylated VLP (Figure 4B) identified four sites of monomannose conjugation (lysines 232, 299, 457 and 562), two of which were also accessible to dimannose conjugation (lysines 232 and 562). As all four lysine residues were localized to the VP60 P (external) domain, which protrudes from the shell (S) domain, it is not surprising that they are accessible for conjugation. The locality of the mannosides on the VP60 P domain also increases the likelihood of recognition and binding by mannose receptors, facilitating mannose associated internalization of the mannosylated VLP.

As further confirmation of the coupling of mannosides **7** and **12** to the VLP, a *Pisum sativum* (mannose selective) lectin blot [35] was performed (Figure 4C). The blot clearly shows an enhanced signal in both mannosylated VLP, confirming mannoside conjugation. The blot also indicates that unmodified VLP carries a low level of glycosylation and this was confirmed with a glycoprotein carbohydrate estimation kit which determined that VLP contained trace amounts of carbohydrate. The mechanism and form of glycosylation is unknown. Carbohydrate estimation was also used to compare the coupling efficiencies of the different mannosides. Comparison of the different VLP with a D-mannose standard provided an estimation of the carbohydrate loading. Subtraction of carbohydrate molarity on unmodified VLP from that of monomannose- and dimannose-VLP provided an estimate 1.5 mole of mannoside per mole of VP60 for both monomannose- and dimannose-VLP. This indicated that although the monomannoside can conjugate to four lysines on each VP60 subunit, only 2 of those (lysines 232 and 562) are readily accessible to modification. As each VLP is composed of 180 copies of VP60, this amounts to as many as 270 copies of each mannoside on each VLP. Therefore, both monomannoside **7** and dimannoside **12** can be successfully conjugated to the surface of VP60, with similar coupling efficiencies, leading to the production of VLP with numerous mannosides projected from their surface.

To allow tracking of VLP uptake by DCs, RHDV VLP was fluorescently labeled with amine-reactive DyLight 633-NHS. To ensure consistent DyLight labeling, and allow direct comparison of the uptake of unmodified VLP, monomannose-VLP and dimannose-VLP, fluorescent labeling was performed before mannoside coupling. DyLight conjugation was performed at a suboptimal molar ratio (1∶1) and reaction time (30 min), to ensure the availability of free lysine residues on the surface of the DyLight labeled VLP for mannoside conjugation. Fluorescence was confirmed by SDS-PAGE (Figure 4D) and quantified by spectrometric analysis, which identified approximately 1 mol of DyLight per mol of VP60 (∼180 copies of DyLight per VLP). Following DyLight labeling, VLP was either left without further modification, or conjugated to an excess of the monomannoside or dimannoside. Mannosylation of DyLight labeled VLP was confirmed by mass-spectrometry, showing conjugation sites consistent with those found in the non-DyLight labeled VLP.

### Testing the Effect of Mannosides on VLP Uptake by murine APCs

**Figure 5:**
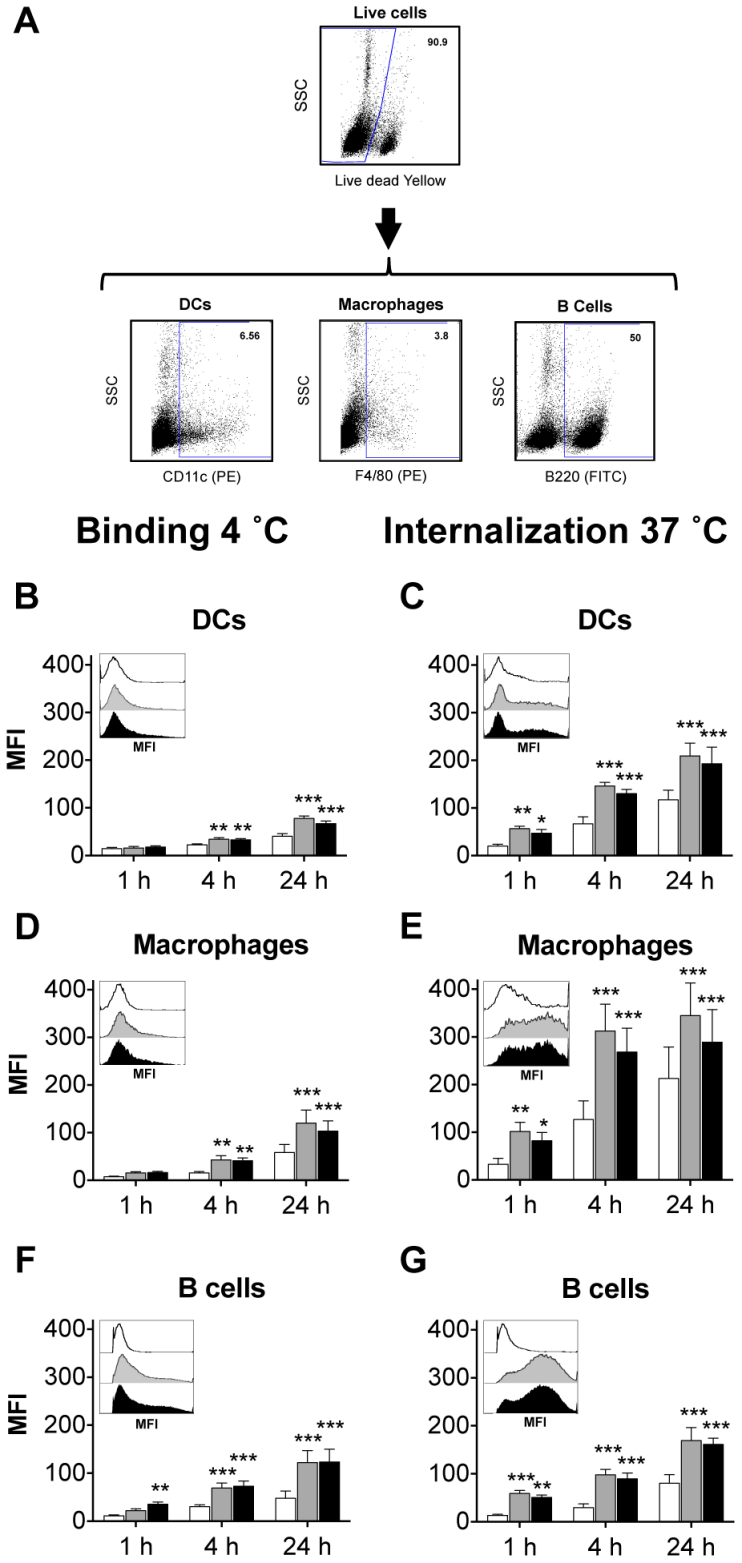
Mannosylation enhances VLP binding and uptake by murine APCs. Murine splenocytes pulsed with DyLight labeled; VLP (white), monomannose-VLP (grey) or dimannose-VLP (black), were incubated at 4°C (B,D,F) or 37°C (C,E,G). Cells were analyzed by flow cytometry to determine the mean fluorescence intensity (MFI) of VLP-DyLight in DCs (B,C), macrophages (D,E) and B cells (F,G). The bar graphs depict the mean MFI from four independent experiments ± S.E.M. Within each figure the MFI of VLP-Dylight in each cell type, at 1 h, is also depicted as a histogram (representative of four experiments). Statistical significance displayed is the comparison between unmodified VLP and monomannose-VLP or dimannose-VLP as determined by matched two-way ANOVA with Bonferroni post-hoc tests, ***p<0.001, **p<0.01.

Multiple professional APCs have the ability to take up RHDV VLP including DCs, macrophages and B cells while non-APCs such as NK cells and T cells do not significantly internalize VLP [4], [10]. Internalization of VLP by APCs is essential for the initiation of the acquired immune response and the generation of immunological memory. Thus, assessment of the effect of mannosylation on VLP uptake was performed *in vitro* with murine DCs, macrophages, B cells and T cells. Cells were pulsed with VLP, monomannose-VLP and dimannose-VLP and incubated for different times at 4°C or 37°C. Cell populations were then analyzed by flow cytometry to compare the different VLP treatments. Figure 5A depicts the gating strategy used in the analysis of VLP binding and uptake assays, whereby following doublet discrimination (not shown), live cells were identified and the different cell populations isolated. The mean fluorescence intensity (MFI) of VLP-DyLight associated with these cells was then determined as an indicator as the amount of VLP bound to, or internalized by the cells. To determine the effect of mannosylation on the association of VLP with the surface of APCs, the interaction of APCs and VLP was studied at 4°C [36], as at this temperature phagocytosis is greatly diminished due to reduced metabolism and membrane fluidity. Analysis of VLP internalization by APCs was performed at 37°C. NK and T cells did not significantly bind or internalize any of the tested VLP (VLP, monomannose-VLP and dimannose-VLP) (data not shown).

DCs and macrophages are two of the major professional antigen presenting cells. They are found throughout the body where they sample the local environment and present foreign antigens to T cells in the lymph nodes and secondary lymphatic organs. DCs and macrophages can process internalized antigens and present them on both MHC class I or class II molecules, and thus are capable of initiating both cytotoxic and helper T cells [12], [37]. DC and macrophages have been shown to express substantial levels of C-type lectins such as, CD206 and DC-SIGN, both of which have a high affinity for mannose and thus have the potential to facilitate enhanced internalization of mannosylated VLP [13]. Examination of VLP association with the surface of DCs and macrophages (4°C) after a 4 or 24-hour incubation indicated that monomannose- and dimannose-VLP displayed augmented association with both cell types compared to unmodified VLP (Figure 5B,D). This enhanced association of mannosylated VLP with DCs and macrophages suggests that mannosylated VLP have the ability to associate with mannose binding receptors on the surface of the APCs. Analysis of VLP uptake (37°C) by DCs and macrophages (Figure 5C,E) depicted an increase in the MFI of monomannose- and dimannose-VLP compared to unmodified VLP in both APCs. This observed difference became more apparent at the later time points (4 and 24 h). These results indicate that mannosylation leads to accelerated uptake of VLP and an increase in the amount of mannosylated and dimannosylated VLP in DCs and macrophages.

Mannosylation of VLP with both mannosides also led to significantly increased binding of VLP to B cells (Figure 5F), professional APCs primarily involved in the generation of humoral immunity. Surprisingly, the binding of mannosylated VLP to B cells at 4°C was found to be even higher than binding to DCs and macrophages (Figure 5B,D). Additionally, an enhanced association of monomannose- and dimannose-VLP with B cells was also observed at 37°C (Figure 5G), although this was relatively low compared to DCs and macrophages (Figure 5B,D). The difference in the dynamics of binding and uptake of mannosylated VLP by B cells and the other two APCs suggests the involvement of an alternate mannose binding receptor. Although B cell expression of C-type lectins and other cell surface receptors has not been as well characterized as that of DCs and macrophages, one group has reported CD206 expression in a subset of B cells [38]. Moreover, B cells express other C-type lectins including; DC immunoreceptors (DCIR), Macrophage-inducible C-type lectin (Mincle) [39], DC associated lectin-1 (DCAL-1) [40] and Dec205 [41]. While the ligand specificity of these receptors is as yet not well characterized, both DCIR-1 and Mincle have both shown selectivity for mannosylated ligands [39]. Furthermore, because numerous pathogens express glycoproteins, glycolipids or thick layers of capsular polysaccharide on their surface, the B1 subset of B cells has evolved to express carbohydrate specific B cell receptors on their cell surface [42], [43]. As the MFI VLP-DyLight in B cells at 4°C and 37°C are similar (Figure 5F,G), it is likely that the VLP is internalized by a non-recycling receptor, such as the B cell receptor.

It is also notable that monomannoside conjugation was found to be at least as efficient, if not significantly better, than dimannoside conjugation at enhancing uptake of VLP by the different APCs (Figure 5B–E). This is surprising, as cellular mannose binding C-type lectins such as DC-SIGN and CD206 have a higher affinity for more complex branched mannosides [23]–[26], [44]. Moreover, White *et. al.* have found that monomannnose conjugation is not sufficient to enhance liposome uptake [45]. This unexpected effectiveness of monomannose may reflect the particulate and repetitive nature of RHDV VLP, as there are four potential sites of mannosylation on each of the 180 VP60 molecules, each protruding at a different angle from the surface of the VLP. This may allow multiple monomannosides to bind to the same receptor, mimicking a more branched mannoside structure for which mannose receptors have a high affinity.

### Confirming the Role of Mannose Recognition in the Binding and Uptake of Mannosylated VLP by murine APCs

**Figure 6:**
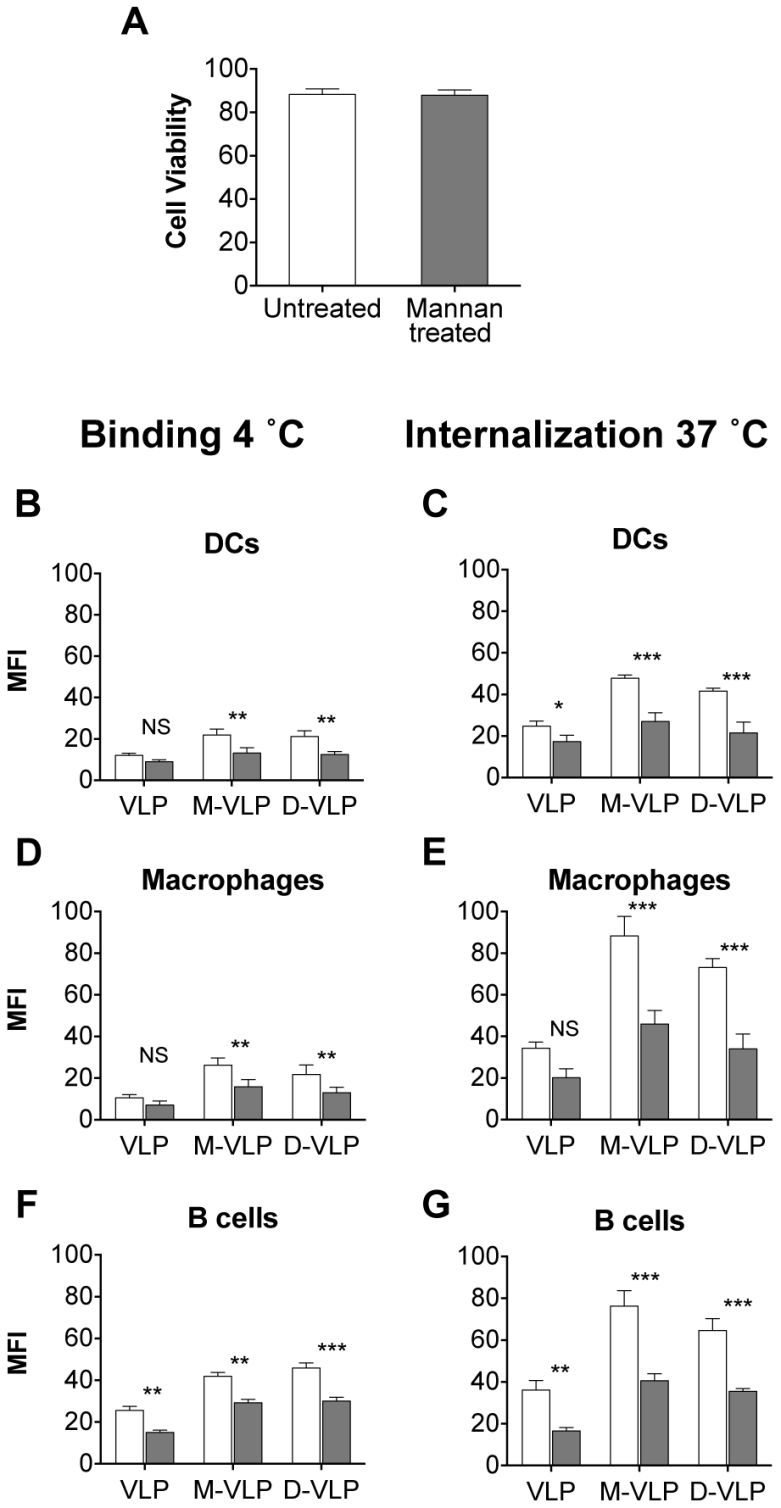
Enhanced mannosylated VLP uptake is due to mannose receptor association. Murine splenocytes were left untreated (white bars) or treated with 3 mg mL^−1^ mannann (grey bars) before a 1 hour incubation with DyLight labeled; VLP, monomannose-VLP (M-VLP) or dimannose-VLP (D-VLP) then analyzed by flow cytometry to determine the mean florescence intensity (MFI) of VLP-DyLight in the different APCs. (A) Cell Viability, as determined by the live dead yellow stain. (B–E) Graphs depict MFI of cells from 3 or 4 independent experiments ± S.E.M. Effect of mannan on VLP binding (4°C) to DCs (B), macrophages (D) and B cells (F). Effect of mannan on the uptake (37°C) of VLP by DCs (C), macrophages (E) and B cells (G). Statistical significance displayed is the comparison between untreated cells and mannan treated cells as determined by matched two-way ANOVA with Bonferroni post-hoc tests, ***p<0.001, **p<0.01, *p<0.05, NS = Non-significant.

Mannan, a polymer of mannose, has been shown to block mannose specific uptake of antigens through competitive binding to mannose receptors [46]–[48]. To confirm whether the augmented binding and uptake of the mannosylated VLP is due to the mannosylation, splenocytes were treated with mannan for 15 min before the addition of the different fluorescently labeled VLP. After a one-hour incubation cell populations were analyzed by flow cytometry to compare the different VLP. 1–3 mg mL^−1^ of mannan has been demonstrated to be effective at mannose receptor inhibition [46], [48], and we found that 5 mg/ml demonstrated no enhanced inhibition (Figure S1) thus 3 mg mL^−1^ of mannan was used in this study. The effect of mannan on cell viability was determined using Fixable live dead yellow stain (Figure 6A), demonstrating no apparent increase in cell death after mannan treatment.

Binding of VLP to the surface of APCs (4°C) showed a mannan dependent reduction in the binding of monomannose- and dimannose-VLP to DCs, macrophages and B cells (Figure 6B,D,F) providing strong evidence that the enhanced binding of mannosylated VLP is due to association with mannose binding receptors. Also notable is a slight but apparent decrease in the binding and uptake of unmodified VLP to these three APCs following mannan treatment (Figure 6B,D,F). This is consistent with an interaction between mannose receptors and the trace amount of carbohydrate detectable on unmodified VLP. Nevertheless, following the addition of monomannoside or dimannoside to the surface of VLP, the interaction between the VLP and mannose receptors was significantly augmented.

Analysis of VLP uptake (37°C) in the presence of mannan showed that the amount of monomannose-VLP and dimannose-VLP in DCs, macrophages and B cells dropped significantly with the addition of mannan (Figure 6C,E,G) as determined by changes in the relative MFI. After the addition of 3 mg mL^−1^ mannan the difference between the uptake of the two mannosylated VLP and unmodified VLP is significantly reduced, confirming the role of mannose recognition in the enhanced uptake of mannosylated VLP in murine DCs, macrophages and B cells.

### The Effect of Mannosides on VLP Uptake by human APCs

**Figure 7:**
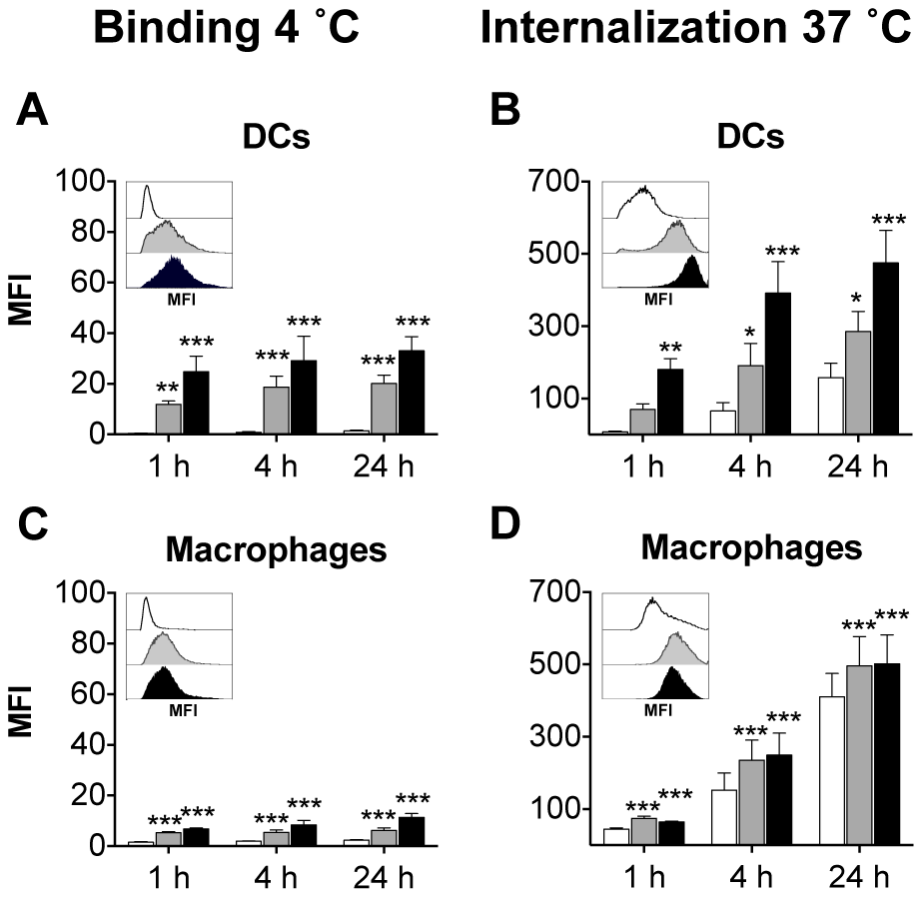
Enhanced binding and internalization of mannosylated VLP is more pronounced in human APCs. Human monocyte-derived DCs and macrophages pulsed with DyLight labeled; VLP (white bars), monomannose-VLP (grey bars) or dimannose-VLP (black bars), were incubated at 4°C (A,C) or 37°C (B,D). Cells were then analyzed by flow cytometry to determine the mean fluorescence intensity (MFI) of VLP-DyLight in DCs (A,B), macrophages (C,D). The graphs depict the mean MFI from four independent experiments ± S.E.M. Within each figure the MFI of VLP-Dylight in each cell type, at 1 h, is also depicted as a histogram (representative of four experiments). Statistical significance displayed is the comparison between unmodified VLP and monomannose-VLP or dimannose-VLP as determined by matched two-way ANOVA with Bonferroni post-hoc tests, ***p<0.001, **p<0.01, *p<0.05.

**Figure 8:**
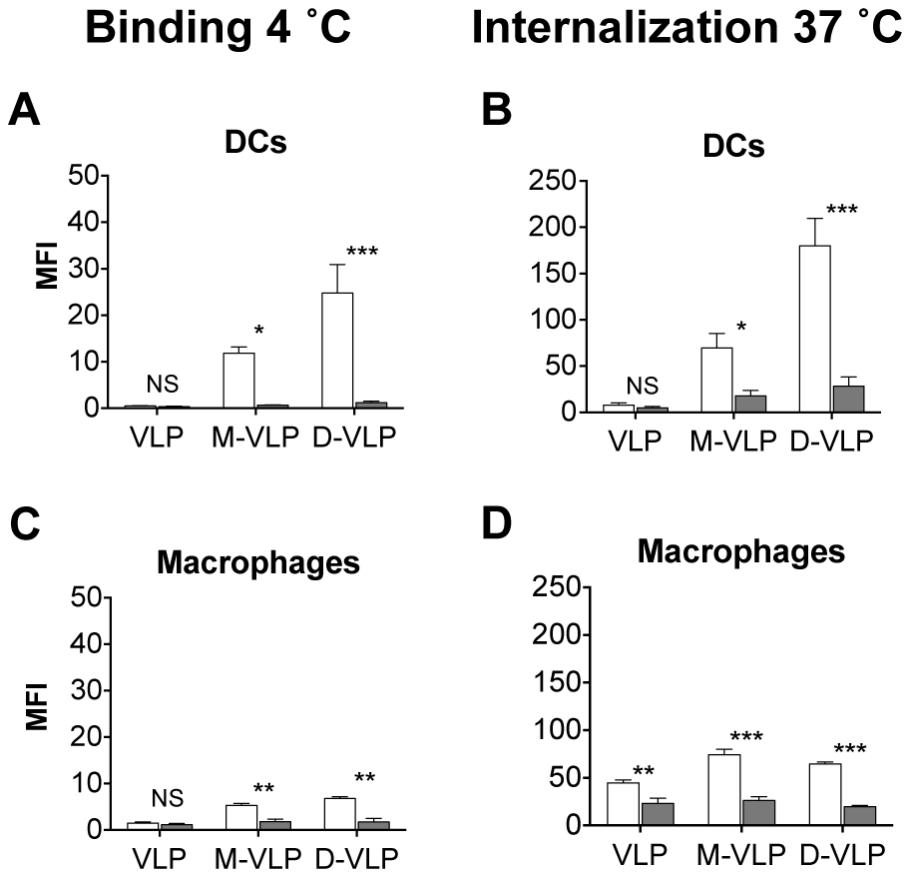
Uptake of mannosylated VLP in human APCs is dependent on mannose receptor association. Human monocyte-derived DCs and macrophages were left untreated (white bars) or treated with 3 mg mL^−1^ mannan (grey bars) before a 1 hour incubation with DyLight labeled; VLP, monomannose-VLP (M-VLP) or dimannose-VLP (D-VLP) then analyzed by flow cytometry to determine the mean florescence intensity (MFI) of VLP-DyLight in the different APCs. (A–D) Graphs depict MFI of cells from four independent experiments ± S.E.M. Effect of mannan on VLP binding (4°C) to DCs (A) and macrophages (C). Effect of mannan on the uptake (37°C) of VLP by DCs (B) and macrophages (D). Statistical significance displayed is the comparison between untreated cells and mannan treated cells as determined by matched two-way ANOVA with Bonferroni post-hoc tests, ***p<0.001, **p<0.01, *p<0.05, NS = Non-significant.

As DCs and Macrophages are the main professional APCs involved in presentation of antigens to cytotoxic T cells, we wished to determine if the enhanced functionality of monomannose- and dimannose-VLP would translate into a human system. Therefore, human monocyte-derived DCs and macrophages were generated and pulsed with the different DyLight labeled VLP at 4°C or 37°C (Figure 7). The MFI of DyLight-VLP was found to be significantly higher for monomannose-VLP and dimannose-VLP compared to unmodified-VLP at both 4°C (Figure 7A,C) and 37°C (Figure 7B,D). This indicates that mannosylation leads to enhanced association and internalization of VLP by human macrophages and DCs. Moreover, following the addition of 3 mg mL^−1^ of mannan to the monocyte-derived DCs and macrophages, the binding (Figure 8A,C) and internalization (Figure 8B,D) of monomannose- and dimannose-VLP was significantly reduced. Confirming the role of mannose recognition in the enhanced uptake of mannosylated VLP in human monocyte-derived DCs and macrophages.

As with murine DCs and macrophages, monomannosylation of VLP significantly enhanced VLP internalization by human APCs. However, unlike murine APCs (Figure 5), human DCs and macrophages bound and internalized dimannose-VLP more efficiently than monomannose-VLP (Figure 7). At 4 and 24 hours, the binding and uptake of dimannose-VLP by DCs (Figure 7A,B) and binding by macrophages (Figure 7C) was found to be significantly higher than that of monomannose-VLP (p<0.01). Differences in the binding affinities of human and mouse APCs to mannosides has been previously noted by White *et. al.* [45] and can by explained by variations in the expression of the different C-type lectins. Another possible explanation is variations within C-type lectin carbohydrate recognition domains, for instance each of the eight carbohydrate recognition domains of CD206 have been found to have 8–24% sequence divergence between the species [44], [49].

### The Effect of Mannosides on VLP cross-presentation by murine BMDCs

**Figure 9:**
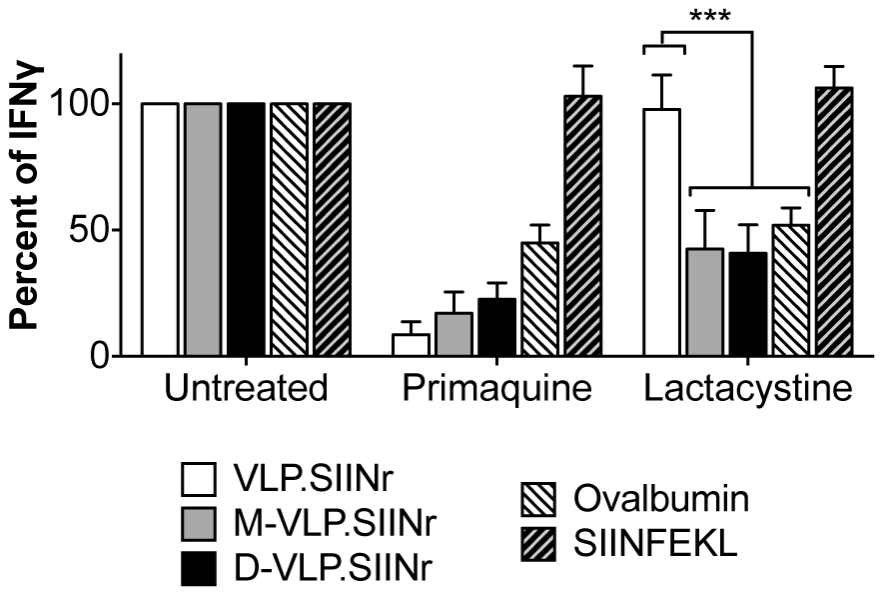
Mannose specific internalization of VLP leads to altered cross-presentation by murine DCs. Murine DCs, pretreated with inhibitors for 15 min, were pulsed with antigen for 24 h before co-culture with OTI T cells. At 72 h IFN-γ levels were measured by enzyme-linked immunosorbent assay (ELISA). Graphs depict the percent IFN-γ produced by inhibitor treated cells relative to untreated cells from three independent experiments performed in duplicate ±S.E.M. Statistical significance determined by matched two-way ANOVA with Bonferroni post-hoc tests, ***p<0.001.

To determine if mannosylation of RHDV VLP alters the processing of VLP, following mannose-associated internalization, DCs were pre-treated with primaquine or lactacystin to block the endosomal recycling or cytosol pathways of cross-presentation respectively, before pulsing with VLP or peptide. DCs were then co-cultured with OT-I splenocytes, and IFN-γ production measured as an indication of CD8 T cell proliferation. Primaquine disengages endosomal trafficking, leading to an inhibition in both the receptor-recycling and to a lesser extent the endosome-to-cytosol pathways of cross-presentation, while lactacystin prevents protein degradation by the proteasome and therefore has minimal effects receptor-recycling pathway. When DC were pulsed with ovalbumin a reduction in SIINFEKL specific CD8+ T-cell activation was observed with both inhibitors, indicating that this protein is processed through a variety of routes, as has been described previously [14](Figure 9). Conversely, the presentation of SIINFEKL peptide was not diminished by either inhibitor (Figure 9), indicating that processing is not essential for effective presentation of free peptides. As expected, a reduction in the cross-presentation of VLP.SIINr was only observed following the pretreatment of DC with primaquine [10] (Figure 9). However, a significant reduction in the cross-presentation of monomannose- and dimannose-VLP.SIINr was observed after the addition of either primaquine or lactacystin. This indicates that mannosylation of RHDV VLP allows the VLP to access an alternative pathway of cross-presentation.

## Conclusions

We have reported the synthesis of a monomannoside and a novel 1,2-α-linked dimannoside, both of which contain an NHS ester allowing conjugation to free lysines at a near neutral pH. Following mannoside conjugation, the VLP retain structural integrity and have up to 270 copies of both mannosides on the surface (P domain) of each particle. These simple mannosides lead to amplified mannose specific binding and internalization of RHDV VLP by murine DCs macrophages and B cells as well as human DCs and macrophages *in vitro*. Unexpectedly, monomannosylation of RHDV VLP was at least as effective as dimannosylation at facilitating mannose specific internalization of VLP by most APCs, although a dimannosylated VLP displayed enhanced functionality in the case of human DCs. The effectiveness of monomannose is probably due to the particulate framework of RHDV VLP allowing the presentation of simple mannosides as branched carbohydrates. Although differences in the binding of mannosylated VLP to the two species of APCs were observed, both the monomannoside and the dimannoside were found to significantly augment VLP internalization in both murine and human APCs. Moreover, mannose specific internalization of RHDV VLP by DCs provides an alternate route of cross-presentation. This demonstrated that the conjugation of mannosides to VLP results in an augmented targeting and delivery system for antigen delivery and an alternate mechanism of intracellular processing.

## Supporting Information

Figure S1 **Titration of Mannan.**
(PDF)

Data S1 **Experimental details for the chemical synthesis of mannosides 1–12.**
(PDF)

Data S2 **^1^H and ^13^C NMR spectra of compounds 3–12.**
(PDF)
