# Supplementary figures and images for: Mannosylation of Virus-Like Particles Enhances Internalization by Antigen Presenting Cells
Source: PLoS One. 2014 Aug 14;9(8):e104523. doi: 10.1371/journal.pone.0104523 (PMC4133192; doi:10.1371/journal.pone.0104523)

Figure S3. Mannan Titration

**Binding 4 °C**

**A**

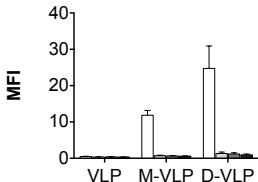

**Internalization 37 °C**

**B**

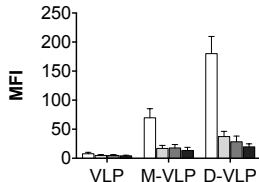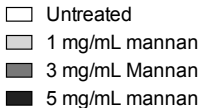

Supplement: Figure S1 — Titration of Mannan. (PDF) [file pone.0104523.s001.pdf]
