# Supplementary material for: Mannosylation of Virus-Like Particles Enhances Internalization by Antigen Presenting Cells
Source: PLoS One. 2014 Aug 14;9(8):e104523. doi: 10.1371/journal.pone.0104523 (PMC4133192; doi:10.1371/journal.pone.0104523)
Supplement: Data S1 — Experimental details for the chemical synthesis of mannosides 1–12. (PDF) [file pone.0104523.s002.pdf]

**Data S1.** Experimental details for the chemical synthesis of mannosides **1** - **12**

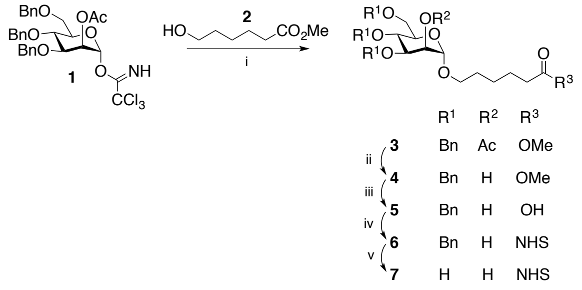


**Synthesis of mannose-NHS (7)**.

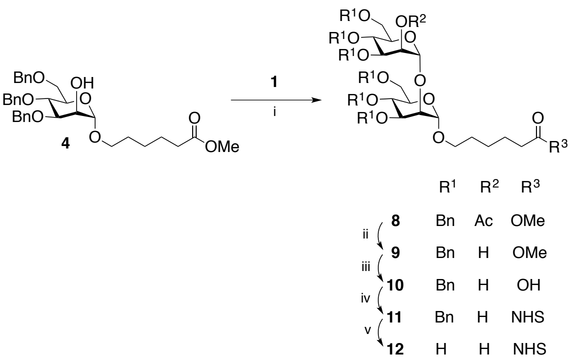


**Synthesis of dimannose-NHS (12)**.

**2-*O*-Acetyl-3,4,6-tri-*O*-benzyl-α-D-mannopyranosyl trichloroacetimidate (1)[1]** Trichloroacetonitrile (2.2 mL, 22.0 mmol) and a catalytic amount of sodium hydride were added to a solution of 2-*O*-acetyl-3,4,6-tri-*O*-benzyl-D-mannopyranose (1.084 g, 2.201 mmol) in dry dichloromethane (10 mL) under nitrogen at 0°C. After stirring for 60 min, the solvent was evaporated under reduced pressure to give the title compound **1**, (1.40 g, quantitative yield). This material was used without further purification. $^{1}$H NMR (400 MHz, $CDCl_3$) δ 2.19 (3H, s, -O(C=O)C$\underline{H}_3$), 3.72 (1H, d, *J* 11.4 Hz, H-6), 3.84 (1H, dd, *J* 12.4 and 4.1 Hz, H-6), 3.96-4.1 (1H, m, H-5), 4.03-4.04 (1H, d, *J* 6.36 Hz, H-4), 4.49-4.88 (6H, m, $PhCH_2$), 5.30 (1H, s, H-3), 5.49 (1H, s, H-2), 6.30 (1H, s, H-1), 7.15-7.36 (15H, m, 3×Ph), 8.36 (1H, s, =NH).

**Methyl 6-hydroxyhexanonate (2)[2]** A 2 % sulfuric acid solution in methanol (50 mL) was added to a solution of ε-caprolactone (5.15 g, 45.1 mmol) in methanol (50 mL). The mixture was heated under reflux for 10 min, then neutralised with sodium carbonate (solid). Excess sodium carbonate was filtered, and the solvent removed under reduced pressure. The mixture was then dissolved in ethyl acetate and washed with water. The organic layer was dried over anhydrous magnesium sulfate, and the solvent removed under reduced pressure. The crude product was purified by Kugelrohr distillation (2 mBar, 150 ˚C), to give the title compound **2** (4.02 g, 27.5 mmol, 61 %). $^1$H NMR (500 MHz, $CDCl_3$) δ 1.37-1.41 (2H, m, H-4), 1.54-1.60 (2H, m, H-5), 1.65 (2H, dt, *J* 15.2 and 7.5 Hz, H-3), 2.32 (2H, t, *J* 7.5, H-2), 3.63 (2H, t, *J* 6.5 Hz, H-6), 3.65 (3H, s, Me); $\nu_{max}$(cm$^{-1}$) = 3413 (broad O-H), 1735 (C=O); HRMS-ESI $[M+Na]^+$ calculated for $C_7H_{14}O_3Na$: 169.0835. Found: 169.0808.

***Methyl 6-[2-O-acetyl-3,4,6-tri-O-benzyl-α-D-mannopyranosyloxy]hexanoate (3)*** Trimethylsilyl triflate (80 µL, 0.442 mmol) was added to a stirring solution of **1** (1.293 g, 2.030 mmol) and **2** (0.386 g, 2.640 mmol) in dry toluene (10 mL) at 0 ˚C, under an inert atmosphere. After an hour triethylamine was added (0.3 mL) and the solvent removed under reduced pressure. The residue was purified by column chromatography (petroleum ether/ethyl acetate 7:1 to 5:1 silica) to give the *title compound* ***3*** (1.030 g, 1.659 mmol, 82 %) as a clear oil. $R_f$= 0.44 (3:1 petroleum ether /ethyl acetate); $[\alpha]_D^{30}$ = +24.6 (*c* 0.9, $CHCl_3$); $^1$H NMR (500 MHz, $CDCl_3$) δ 1.32-1.38 (2H, m, H-4), 1.54-1.59 (3H, m, H-5), 1.63 (2H, quin, *J* 7.7 Hz, H-3), 2.15 (3H, s, Ac C<u>H</u>$_3$), 2.31 (2H, t, *J* 7.5 Hz, H-2), 3.40 (1H, dt, *J* 6.5 and 9.6 Hz, H-6), 3.65 (3H, s, Me C<u>H</u>$_3$ ), 3.67-3.71 (2H, m, H-6, H-6’), 3.76-3.81 (2H, m, H-5’, H-6’), 3.88 (1H, t, *J* 9.4 Hz, H-4’), 3.97 (1H, dd, *J* 3.3 and 9.3 Hz, H-3’), 4.47 (1H, d, *J* 10.7 Hz, PhC<u>H</u>$_2$), 4.52 (1H, d, *J* 12.4 Hz, PhC<u>H</u>$_2$), 4.54 (1H, d, *J* 10.9 Hz, PhC<u>H</u>$_2$), 4.68 (1H, d, *J* 13.2 Hz, PhC<u>H</u>$_2$), 4.71 (1H, d, *J* 10.6 Hz, PhC<u>H</u>$_2$), 4.82 (1H, d, *J* 1.4 Hz, H-1’), 4.85 (1H, d, *J* 10.7 Hz, PhC<u>H</u>$_2$), 5.35 (1H, dd, *J* 1.8 and 3.2 Hz, H-2’), 7.15-7.36 (13H, m, Ph); $^{13}$C NMR (125 MHz, $CDCl_3$) δ 21.30 (Ac $CH_3$), 24.85 (C-3), 25.87 (C-4), 29.21 (C-5), 34.09 (C-2), 51.63 (Me $CH_3$), 67.82 (C-6), 69.02 (C-2’), 69.05 (C-6’), 71.53 (C-5’), 71.95 (Ph), 73.59 (Ph), 74.52 (C-4’), 75.37 (Ph), 78.41 (C-3’), 97.91 ($^1J_{CH}$ 169.8 Hz, C-1’), 127.72 (Ph), 127.79 (Ph), 127.87 (Ph), 127.92 (Ph), 128.09 (Ph), 128.21 (Ph), 128.45 (Ph), 128.47 (Ph), 128.53 (Ph), 138.14 (Ph), 138.39 (Ph), 138.50 (Ph), 170.69 (Ac C=O), 174.19 (C-1); $\nu_{max}$(cm$^{-1}$) = 3063 (arom. C-H), 3030 (arom. C-

H), 2927 (C-H), 2865 (C-H), 1737 (C=O); HRMS-ESI $[M+Na]^+$ calculated for $C_{36}H_{44}O_9Na$: 643.2878. Found: 643.2869.

**Methyl 6-[3,4,6-tri-*O*-benzyl-α-D-mannopyranosyloxy]hexanoate (4)[3]** Aqueous sodium methoxide in methanol (0.236 M, 20 mL) was added to a solution of **3** (0.586 g, 0.944 mmol) in dichloromethane (20 mL) and stirred for an hour. The mixture was then dissolved in ethyl acetate and washed with water. The organic layer was dried over anhydrous magnesium sulfate, and the solvent removed under reduced pressure. The crude product was purified by column chromatography (petroleum ether/ethyl acetate 2:1 silica) to give the title compound **4** (0.382 g, 0.660 mmol, 70 %) as a clear oil. $R_f$= 0.75 (3:1 dichloromethane/ethyl acetate); $[\alpha]_D^{24}$ = +39.1 (*c* 1.46, $CHCl_3$) [lit.[3] $[\alpha]_D^{25}$ = +36 (*c* 0.8, $CHCl_3$)]; $^1$H NMR (500 MHz, $CDCl_3$) δ 1.33-1.38 (2H, m, H-4), 1.54-1.60 (2H, m, H-5), 1.63 (3H, quin , *J* 7.6 Hz, H-3), 2.30 (2H, t, *J* 7.5 Hz, H-2), 3.41 (1H, dt, *J* 6.5 and 9.7 Hz, H-6), 3.65 (3H, s, Me $C\underline{H}_3$ ), 3.66-3.77 (4H, m, H-6, H-5', H-6'), 3.83 (1H, t, *J* 7.5 Hz, H-4'), 3.88 (1H, dd, *J* 3.1 and 9.0 Hz, H-3'). 4.02 (1H, dd, *J* 1.8 and 3.1 Hz, H-2'), 4.50 (1H, d, *J* 10.8 Hz, $PhC\underline{H}_2$), 4.54 (1H, d, *J* 12.2 Hz, $PhC\underline{H}_2$), 4.65 (1H, d, *J* 12.2 Hz, $PhC\underline{H}_2$), 4.68 (1H, d, *J* 11.4 Hz, $PhC\underline{H}_2$), 4.72 (1H, d, *J* 11.4 Hz, $PhC\underline{H}_2$), 4.82 (1H, d, *J* 10.8 Hz, $PhC\underline{H}_2$), 4.88 (1H, d, *J* 1.7 Hz, H-1'), 7.16-7.38 (13H, m, Ph); $^{13}$C NMR (125 MHz, $CDCl_3$) δ 24.85 (C-3), 25.89 (C-4), 29.22 (C-5), 34.11 (C-2), 51.63 ($CH_3$), 67.60 (C-6), 68.59 (C-2'), 69.11 (C-6'), 71.20 (C-5'), 72.13 (Ph), 73.60 (Ph), 74.49 (C-4'), 75.32 (Ph), 80.45 (C-3'), 99.32 (C-1'), 127.69 (Ph), 127.83 (Ph), 127.96 (Ph), 127.98 (Ph), 128.04 (Ph), 128.13 (Ph), 128.45 (Ph), 128.50 (Ph), 128.66 (Ph), 138.11 (Ph), 138.41 (Ph), 138.42 (Ph), 174.19 (C-1); $\nu_{max}(cm^{-1})$ = 3451 (broad O-H), 3063 (arom. C-H), 3029 (arom. C-H), 2914 (C-H), 2864 (C-H), 1735 (C=O); HRMS-ESI $[M+Na]^+$ calculated for $C_{34}H_{42}O_8Na$: 601.2772. Found: 601.2790.

***6-[3,4,6-Tri-O-benzyl-α-D-mannopyranosyloxy]hexanoic acid (5)*** Sodium hydroxide (1 M, 3.31 mL) was added to a solution of **4** (0.320 g, 0.553 mmol) in THF (10 mL) and stirred for 24 hours at 60 °C. The solution was neutralised with Amyberlyst 15 ($H^+$) ion exchange resin, then filtered and the solvent removed *in vacuo*. The residue was purified by column chromatography (petroleum ether/ethyl acetate 3:1 silica) to give the *title compound* ***5*** (0.281 g, 0.498 mmol, 90 %) as a clear oil. $R_f$= 0.53 (3:1 dichloromethane/ethyl acetate); $[\alpha]_D^{30}$ = +38.9 (*c* 1.0, $CHCl_3$); $^1$H NMR (500 MHz, $CDCl_3$) δ 1.38 (2H, quin, *J* 7.7 Hz, H-4), 1.55-1.59 (2H, m, H-5), 1.64 (2H, quin, *J* 7.5 Hz, H-3), 2.33 (2H, t, *J* 7.4 Hz, H-2), 3.41 (1H, dt, *J* 6.4 and 9.6 Hz, H-6), 3.66-

3.78 (4H, m, H-6, H-5', H-6'), 3.84 (1H, t, *J* 9.2 Hz, H-4'), 3.88 (1H, dd, *J* 3.1 and 9.0 Hz, H-3'), 4.02 (1H, d, *J* 1.6 Hz, H-2'), 4.50 (1H, d, *J* 10.8 Hz, PhC$\underline{H}_2$), 4.54 (1H, d, *J* 12.2 Hz, PhC$\underline{H}_2$), 4.65 (1H, d, *J* 12.2 Hz, PhC$\underline{H}_2$), 4.68 (1H, d, *J* 11.5 Hz, PhC$\underline{H}_2$), 4.72 (1H, d, *J* 11.4 Hz, PhC$\underline{H}_2$), 4.82 (1H, d, *J* 10.8 Hz, PhC$\underline{H}_2$), 4.88 (1H, s, H-1'), 7.16-7.38 (14H, m, Ph); $^{13}$C NMR (125 MHz, $CDCl_3$) δ 24.53 (C-3), 25.75 (C-4), 29.12 (C-5), 33.95 (C-2), 67.51 (C-6), 68.54 (C-2'), 69.06 (C-6'), 71.18 (C-5'), 72.10 (Ph), 73.56 (Ph), 74.47 (C-4'), 75.30 (Ph), 80.39 (C-3'), 99.31 (C-1'), 127.68 (Ph), 127.82 (Ph), 127.97 (Ph), 127.98 (Ph), 128.02 (Ph), 128.11 (Ph), 128.43 (Ph), 128.48 (Ph), 128.62 (Ph), 138.06 (Ph), 138.33 (Ph), 138.36 (Ph), 179.18 (C-1); $\nu_{max}$(cm$^{-1}$) = 3443 (broad O-H), 3063 (arom. C-H), 3030 (arom. C-H), 2920 (C-H), 2865 (C-H), 1706 (C=O); HRMS-ESI [M+Na]$^+$ calculated for $C_{33}H_{40}O_8Na$: 587.2615. Found: 587.2577.

***N-Succinimidyl 6-[3,4,6-tri-O-benzyl-α-D-mannopyranosyloxy]hexanoate (6)***

N,N-Dicyclohexylcarbodiimide (0.067 g, 0.324 mmol) and N-hydroxysuccinimide (0.037 g, 0.324 mmol) were added to **5** ( 0.055 g, 0.097 mmol) in THF (3 mL), and stirred overnight. The solvent was removed *in vacuo* and crude product was purified by column chromatography (dichloromethane/ethyl acetate 4:1 silica) to give the title compound **6** (0.064 g, quantitative yield) as a clear oil, containing a trace of N,N-dicyclohexylurea (DCU). A small sample was further purified by column chromatography (dichloromethane to dichloromethane/ethyl acetate 9:1 to 4:1 silica) to give the *title compound* **6** as a clear oil. $R_f$ = 0.61 (4:1 dichloromethane/ethyl acetate); $[\alpha]_D^{32}$ = +32.4 (*c* 1.0, $CHCl_3$); $^1$H NMR (500 MHz, $CDCl_3$) δ 1.46 (2H, quin, *J* 7.7 Hz, H-4), 1.57-1.63 (2H, m, H-5), 1.73-1.79 (2H, m, H-3), 2.60 (2H, t, *J* 7.4 Hz, H-2), 2.76 (4H, s, NHS 2×C$\underline{H}_2$), 3.43 (1H, dt, *J* 6.3 and 9.8 Hz, H-6), 3.68-3.78 (4H, m, H-6, H-5', H-6'), 3.84 (1H, t, *J* 9.1 Hz, H-4'), 3.89 (1H, dd, *J* 3.1 and 9.0 Hz, H-3'), 4.04 (1H, dt, *J* 7.7 and 15.1 Hz, H-2'), 4.50 (1H, d, *J* 10.8 Hz, PhC$\underline{H}_2$), 4.54 (1H, d, *J* 12.1 Hz, PhC$\underline{H}_2$), 4.65 (1H, d, *J* 12.1 Hz, PhC$\underline{H}_2$), 4.69 (1H, d, *J* 11.5 Hz, PhC$\underline{H}_2$), 4.72 (1H, d, *J* 11.5 Hz, PhC$\underline{H}_2$), 4.81 (1H, d, *J* 10.8 Hz, PhC$\underline{H}_2$), 4.88 (1H, s, H-1'), 7.16-7.38 (15H, m, Ph); $^{13}$C NMR (125 MHz, $CDCl_3$) δ 24.52 (C-3), 25.56 (C-4), 25.68 (NHS $CH_2$), 29.02 (C-5), 31.04 (C-2), 67.40 (C-6), 68.57 (C-2'), 69.10 (C-6'), 71.20 (C-5'), 72.08 (Ph), 73.58 (Ph), 74.49 (C-4'), 75.27 (Ph), 80.39 (C-3'), 99.38 (C-1'), 127.68 (Ph), 127.80 (Ph), 127.96 (Ph), 127.99 (Ph), 128.00 (Ph), 128.10 (Ph), 128.44 (Ph), 128.48 (Ph), 128.64 (Ph), 138.18 (Ph), 138.40 (Ph), 138.47 (Ph), 168.62 (C-1), 169.26 (NHS C=O); $\nu_{max}$(cm$^{-1}$) = 3503 (broad O-H), 3063 (arom.

C-H), 3030 (arom. C-H), 2920 (C-H), 2866 (C-H), 1812 (imide C=O), 1783 (imide C=O), 1735 (C=O); HRMS-ESI $[M+Na]^+$ calculated for $C_{37}H_{43}NO_{10}Na$: 684.2779. Found: 684.2756.

**N-Succinimidyl 6-[α-D-mannopyranosyloxy]hexanoate (7)[4]** 20% $Pd(OH)_2/C$ catalyst (0.085 g) was added to a solution of **6** (containing DCU contamination) (0.064 g) in ethyl acetate (7 mL). The reaction was stirred overnight under hydrogen, filtered through celite, and the crude product purified by column chromatography ($CHCl_3$/MeOH 5:1 silica) to give the title compound **7** (0.029 mg, 0.074 mmol, 77 %) as a white powder. $R_f$= 0.40 (5:1 $CHCl_3$/MeOH); $[\alpha]_D^{23}$ = +47.3 (*c* 1.0, acetone); $^1$H NMR (500 MHz, acetone-$d_6$) δ 1.51-1.55 (2H, m, H-4), 1.61-1.67 (2H, m, H-5), 1.72-1.79 (2H, m, H-3), 2.65 (2H, t, *J* 7.3 Hz, H-2), 2.87 (4H, s, NHS 2×C$\underline{H}_2$), 3.42 (1H, dt, *J* 6.2 and 9.7 Hz, H-6), 3.52 (1H, ddd, *J* 3.0, 5.7 and 8.8 Hz, H-5'), 3.67-3.70 (3H, m, H-3', H-4', H-6'), 3.73 (1H, dt, *J* 6.5, 9.7 Hz, H-6), 3.77-3.79 (2H, m, H-2', H-6'), 4.75 (1H, d, *J* 1.3 Hz, H-1'); $^{13}$C NMR (125 MHz, acetone-$d_6$) δ 25.22 (C-3), 26.11 (C-4), 26.27 (NHS $CH_2$), 29.71 (C-5), 31.23 (C-2), 62.99 (C-6'), 67.54 (C-6), 68.90 (C-4'), 71.83 (C-2'), 72.67 (C-3'), 73.99 (C-5'), 101.01 (C-1'), 169.63 (C-1), 170.60 (NHS C=O); $\nu_{max}(cm^{-1})$ = 3378 (broad O-H), 2935 (C-H), 1812 (imide C=O), 1781 (imide C=O), 1729 (C=O); HRMS-ESI $[M+Na]^+$ calculated for $C_{16}H_{25}NO_{10}Na$: 414.1371. Found: 414.1378.

***Methyl 6-[2-O-acetyl-3,4,6-tri-O-benzyl-2-O-(3,4,6-tri-O-benzyl-α-D-mannopyranosyl)-α-D-mannopyranosyloxy]hexanoate (8)*** Trimethylsilyl triflate (22.6 µL, 0.208 mmol) was added to a stirring solution of **1** (0.663 g, 1.040 mmol) and **4** (0.400 g, 0.694 mmol) in dry dichloromethane (10 mL) cooled to 0 ˚C, under an inert atmosphere. After 1 hour triethylamine was added (0.1 mL) and the solvent removed under reduced pressure. The crude product was purified by column chromatography (petroleum ether/ethyl acetate 9:1 to 5:1 silica) to give the *title compound* ***8*** (0.650 g, 0.617 mmol, 89 %) as a clear oil. $R_f$= 0.39 (3:1 petroleum ether/ethyl acetate); $[\alpha]_D^{23}$ = +23.33 (*c* 0.9, $CHCl_3$); $^1$H NMR (500 MHz, $CDCl_3$) δ 1.26-1.32 (2H, m, H-4), 1.47-1.53 (2H, m, H-5), 1.60 (2H, quin, *J* 7.6 Hz, H-3), 2.12 (3H, s, Ac C$\underline{H}_3$), 2.28 (2H, t, *J* 7.5 Hz, H-2), 3.25 (1H, dt, *J* 6.6 and 9.1 Hz, H-6), 3.58 (1H, dt, *J* 6.6 and 9.1 Hz, H-6), 3.64 (3H, s, Me C$\underline{H}_3$ ), 3.68-3.81 (6H, m, H-4', H-5', H-6', H-6"), 3.84 (1H, dd, *J* 3.3 and 9.3 Hz, H-4"), 3.90 (1H, dd, *J* 2.7 and 9.3 Hz, H-3'), 3.95-3.99 (3H, m, H-2', H-3", H-5"), 4.39-4.83 (12H, m, PhC$\underline{H}_2$), 4.86 (1H, s, H-1'), 5.08 (1H, s, H-1"), 5.54 (1H, s, H-2"), 7.15-7.34 (22H, m, Ph); $^{13}$C NMR (125

MHz, $CDCl_3$) δ 21.28 (Ac $CH_3$), 24.86 (C-3), 25.87 (C-4), 29.26 (C-5), 34.10 (C-2), 51.61 (Me $CH_3$), 67.56 (C-6), 68.89 (C-2''), 69.25 and 69.44 (C-6' and C-6'')*, 71.93 (C-5'), 71.98 (C-5''), 72.05 (Ph), 72.17 (Ph), 73.43 (Ph), 73.53 (Ph), 74.53 and 74.82 (C-4' and C-4'')*, 75.13 (C-2'), 75.19 (Ph), 75.33 (Ph), 78.28 (C-3''), 79.87 (C-3'), 98.82 ($^1J_{CH}$ 170.1 Hz, C-1'), 99.7 ($^1J_{CH}$ 169.8 Hz, C-1''), 127.53 (Ph), 127.61 (Ph), 127.63 (Ph), 127.65 (Ph), 127.67 (Ph), 127.70 (Ph), 127.77 (Ph), 127.78 (Ph), 127.91 (Ph), 128.24 (Ph), 128.30 (Ph), 128.40 (Ph), 128.41 (Ph), 128.42 (Ph), 128.46 (Ph), 128.47 (Ph), 128.51 (Ph), 138.15 (Ph), 138.36 (Ph), 138.53 (Ph), 138.55 (Ph), 138.62 (Ph), 138.68 (Ph), 170.26 (Ac C=O), 174.18 (C-1); $\nu_{max}(cm^{-1})$ = 3063 (arom. C-H), 3029 (arom. C-H), 2917 (C-H), 2864 (C-H), 1737 (C=O); HRMS-ESI $[M+Na]^+$ calculated for $C_{63}H_{72}O_{14}Na$: 1075.4814. Found: 1075.4796

**Methyl 6-[3,4,6-tri-*O*-benzyl-2-*O*-(3,4,6-tri-*O*-benzyl-α-D-mannopyranosyl)-α-D-mannopyranosyloxy]hexanoate (9)[3]** Sodium methoxide (0.250 M) in methanol (12 mL) was added to a solution of **8** (0.626 g, 0.594 mmol) in dichloromethane (12 mL) and stirred for an hour. The mixture was then dissolved in ethyl acetate and washed with water. The organic layer was dried over anhydrous magnesium sulfate, and the solvent removed under reduced pressure. The crude product was purified by column chromatography (dichloromethane/ethyl acetate 3:1 silica) to give the title compound **9** (0.550 g, 0.544 mmol, 92 %) as a clear oil. $R_f$ = 0.83 (3:1 dichloromethane/ethyl acetate); $[\alpha]_D^{23}$ = +32.0 (*c* 1.7, $CHCl_3$) [lit.[3] $[\alpha]_D^{25}$ = +34 (*c* 0.5, $CHCl_3$)]; $^1$H NMR (500 MHz, $CDCl_3$) δ 1.24-1.31 (3H, m, H-4), 1.46-1.52 (2H, m, H-5), 1.59 (2H, quin, *J* 7.6 Hz, H-3), 2.28 (2H, t, *J* 7.6 Hz, H-2), 3.24 (1H, dt, *J* 6.5 and 9.6 Hz, H-6), 3.57 (1H, dt, *J* 6.7 and 9.6 Hz, H-6), 3.65 (3H, s, Me C$\underline{H}_3$ ), 3.69-3.84 (7H, m, H-4', H-5', H-6', H-4'', H-6''), 3.86 (1H, dd, *J* 3.5 and 6.9 Hz, H-3''), 3.91 (1H, dd, *J* 2.9 and 9.3 Hz, H-3'), 3.96 (1H, ddd, *J* 2.3, 4.7 and 9.8 Hz, H-5''), 4.01 (1H, dd, *J* 2.1 and 2.7 Hz, H-2'), 4.12 (1H, dd, *J* 1.8 and 3.2 Hz, H-2''), 4.48-4.84 (12H, m, PhC$\underline{H}_2$), 4.89 (1H, d, *J* 1.8 Hz, H-1'), 5.14 (1H, d, *J* 1.6 Hz, H-1''), 7.17-7.34 (25H, m, Ph);$^{13}$C NMR (125 MHz, $CDCl_3$) δ 24.85 (C-3), 25.86 (C-4), 29.26 (C-5), 34.08 (C-2), 51.59 (Me $CH_3$), 67.55 (C-6), 68.66 (C-2''), 69.36 and 69.46 (C-6' and C-6'')*, 71.65 (C-5''), 72.01 (C-5'), 72.26 (Ph), 72.38 (Ph), 73.42 (Ph), 73.52 (Ph), 74.56 and 74.96 (C-4' and C-4'')*, 75.13 (C-2), 75.18 (Ph), 75.29 (Ph), 79.93 (C-3'), 80.13 (C-3''), 98.93 (C-1'), 101.21 (C-1''), 127.48 (Ph), 127.56 (Ph), 127.65 (Ph), 127.71 (Ph), 127.77 (Ph), 127.80 (Ph), 127.85 (Ph), 127.94 (Ph), 127.95 (Ph), 127.99 (Ph), 128.13 (Ph), 128.39 (Ph), 128.40 (Ph), 128.43 (Ph), 128.47 (Ph),

128.56 (Ph), 128.58 (Ph), 138.12 (Ph), 138.38 (Ph), 138.45 (Ph), 138.50 (Ph), 138.57 (Ph), 138.76 (Ph), 174.17 (C-1); $\nu_{max}(cm^{-1})$ = 3473 (broad O-H), 3063 (arom. C-H), 3030 (arom. C-H), 2912 (C-H), 2864 (C-H), 1736 (C=O); HRMS-ESI $[M+Na]^+$ calculated for $C_{61}H_{70}O_{13}Na$: 1033.4709. Found: 1033.4702.

**6-[3,4,6-Tri-*O*-benzyl-2-*O*-(3,4,6-tri-*O*-benzyl-α-D-mannopyranosyl)-α-D-mannopyranosyloxy]hexanoic acid (10)[3]** Aqueous sodium hydroxide (1 M, 3.02 mL) was added to a solution of **9** (0.510 g, 0.504 mmol) in THF (9 mL) and stirred for 24 hours at 60 °C. The solution neutralised with Amberlyst 15 ($H^+$) ion exchange resin, then filtered and the solvent removed *in vacuo*. The crude product was purified by column chromatography (dichloromethane/ethyl acetate 3:1 silica) to give the title compound **10** (0.430 g, 0.431 mmol, 85 %) as a clear oil. $R_f$ = 0.74 (3:1 dichloromethane/ethyl acetate); $[\alpha]_D^{22}$ = +31.0 (*c* 1.1, $CHCl_3$) [lit.[3] $[\alpha]_D^{25}$ = +36 (*c* 0.5, $CHCl_3$)]; $^1$H NMR (500 MHz, $CDCl_3$) δ 1.28-1.35 (2H, m, H-4), 1.48-1.54 (2H, m, H-5), 1.61 (2H, quin, *J* 7.4 Hz, H-3), 2.31 (2H, t, *J* 7.4 Hz, H-2), 3.27 (1H, dt, *J* 6.3 and 9.2 Hz, H-6), 3.59 (1H, dt, *J* 6.5 and 9.2 Hz, H-6), 3.71-3.87 (7H, m, H-4', H-5', H-6', H-4", H-6"), 3.90 (1H, dd, *J* 2.4 and 8.9 Hz, H-3"), 3.94 (1H, dd, *J* 2.4 and 9.4 Hz, H-3'), 3.97-3.99 (1H, m, H-5"), 4.04 (1H, s, H-2'), 4.15 (1H, s, H-2"), 4.51-4.87 (12H, m, $PhC\underline{H_2}$), 4.92 (1H, s, H-1'), 5.18 (1H, s, H-1"), 7.18-7.36 (30H, m, Ph); $^{13}$C NMR (125 MHz, $CDCl_3$) δ 24.54 (C-3), 25.68 (C-4), 29.12 (C-5), 33.91 (C-2), 67.39 (C-6), 68.65 (C-2"), 69.37 and 69.45 (C-6' and C-6")*, 71.64 (C-5"), 71.98 (C-5'), 72.26 (Ph), 72.38 (Ph), 73.41 (Ph), 73.51 (Ph), 74.57 and 74.96 (C-4' and C-4")*, 75.06 (C-2), 75.15 (Ph), 75.29 (Ph), 79.90 (C-3'), 80.12 (C-3"), 98.88 (C-1'), 101.15 (C-1"), 127.49 (Ph), 127.57 (Ph), 127.66 (Ph), 127.73 (Ph), 127.77 (Ph), 127.80 (Ph), 127.88 (Ph), 127.97 (Ph), 127.99 (Ph), 128.01 (Ph), 128.12 (Ph), 128.38 (Ph), 128.41 (Ph), 128.43 (Ph), 128.47 (Ph), 128.55 (Ph), 128.58 (Ph), 138.08 (Ph), 138.32 (Ph), 138.42 (Ph), 138.44 (Ph), 138.51 (Ph), 138.71 (Ph), 178.67 (C-1); $\nu_{max}(cm^{-1})$ = 3443 (broad O-H), 3063 (arom. C-H), 3030 (arom. C-H), 2921 (C-H), 2865 (C-H), 1705 (C=O); HRMS-ESI $[M+Na]^+$ calculated for $C_{60}H_{68}O_{13}Na$: 1019.4552. Found: 1019.4531.

***N-Succinimidyl 6-[3,4,6-tri-O-benzyl-2-O-(3,4,6-tri-O-benzyl-α-D-mannopyranosyl)-α-D-mannopyranosyloxy]hexanoate (11)*** N,N-Dicyclohexylcarbodiimide (0.046 g, 0.224 mmol) and N-hydroxysuccinimide (0.026 g, 0.224 mmol) were added to **10** (0.067 g, 0.067 mmol) in THF (3 mL), and stirred overnight. The solvent was removed *in vacuo* and crude product was purified by column chromatography

(dichloromethane/ethyl acetate 4:1 silica) to give the *title compound* ***11*** (0.074 g, quantitative yield) as clear oil, containing a trace of N,N-dicyclohexylurea (DCU). A small sample was further purified by column chromatography (dichloromethane to dichloromethane/ethyl acetate 9:1 to 4:1 silica) to give the *title compound* ***11*** as a foam. $R_f$= 0.88 (4:1 dichloromethane/ethyl acetate); $[\alpha]_D^{23}$ = +26.4 (*c* 0.7, $CHCl_3$); $^1$H NMR (500 MHz, $CDCl_3$) δ 1.36-1.42 (2H, m, H-4), 1.49-1.55 (2H, m, H-5), 1.71 (2H, quin, *J* 7.4 Hz, H-3), 2.56 (2H, t, *J* 7.4 Hz, H-2), 2.74 (4H, s, NHS 2xC$\underline{H}_2$), 3.26 (1H, dt, *J* 6.3 and 9.6 Hz, H-6), 3.59 (1H, dt, *J* 6.4 and 9.5 Hz, H-6), 3.70-3.85 (7H, m, H-4', H-5', H-6', H-4", H-6"), 3.87 (1H, dd, *J* 3.0 and 9.1 Hz, H-3"), 3.93 (1H, dd, *J* 2.8 and 9.4 Hz, H-3'), 3.96 (1H, ddd, *J* 2.1, 4.4 and 9.7 Hz, H-5"), 4.03 (1H, s, H-2'), 4.13 (1H, s, H-2"), 4.49-4.84 (12H, m, PhC$\underline{H}_2$), 4.90 (1H, s, H-1'), 5.14 (1H, s, H-1"), 7.17-7.34 (26H, m, Ph); $^{13}$C NMR (125 MHz, $CDCl_3$) δ 24.53 (C-3), 25.54 (C-4), 25.68 (NHS $CH_2$) 29.05 (C-5), 31.05 (C-2), 67.37 (C-6), 68.69 (C-2"), 69.38 and 69.49 (C-6' and C-6")*, 71.65 (C-5"), 72.03 (C-5'), 72.27 (Ph), 72.35 (Ph), 73.42 (Ph), 73.53 (Ph), 74.58 and 74.99 (C-4' and C-4")*, 75.13 (C-2), 75.24 (Ph), 75.26 (Ph), 79.91 (C-3'), 80.15 (C-3"), 98.98 (C-1'), 101.24 (C-1"), 127.49 (Ph), 127.58 (Ph), 127.67 (Ph), 127.72 (Ph), 127.76 (Ph), 127.80 (Ph), 127.86 (Ph), 127.97 (Ph), 127.98 (Ph), 128.00 (Ph), 128.12 (Ph), 128.40 (Ph), 128.43 (Ph), 128.44 (Ph), 128.48 (Ph), 128.57 (Ph), 128.60 (Ph), 138.14 (Ph), 138.42 (Ph), 138.54 (Ph), 138.54 (Ph), 138.65 (Ph), 138.79 (Ph), 168.63 (C-1), 169.27 (NHS C=O); $\nu_{max}$(cm$^{-1}$) = 3509 (broad O-H), 3063 (arom. C-H), 3030 (arom. C-H), 2920 (C-H), 2866 (C-H), 1813 (imide C=O), 1783 (imide C=O), 1737 (C=O); HRMS-ESI $[M+Na]^+$ calculated for $C_{64}H_{71}NO_{15}Na$: 1019.4552. Found: 1019.4531.

***N-Succinimidyl 6-[2-O-(α-D-mannopyranosyl)-α-D-mannopyranosyloxy] hexanoate (12)*** $Pd(OH)_2$/C catalyst (0.080 g) was added to a solution of **11** (0.074 g) in THF (10 mL). The reaction was stirred overnight under hydrogen. The mixture was filtered through Celite, and the crude product was purified by column chromatography ($CHCl_3$/MeOH 7:4 silica) to give the *title compound* ***12*** (0.028 g, 0.051 mmol, 75 %) as a white powder. $R_f$= 0.47 (7:4 $CHCl_3$/MeOH); $[\alpha]_D^{25}$ = +56.0 (*c* 1.0, methanol); $^1$H NMR (500 MHz, methanol-*$d_6$*) δ 1.50-1.55 (2H, m, H-4), 1.61-1.67 (2H, m, H-5), 1.77 (2H, quin, *J* 7.6 Hz, H-3), 2.65 (2H, t, *J* 7.3 Hz, H-2), 2.83 (4H, s, NHS 2xC$\underline{H}_2$), 3.43-3.61 (4H, m, H-6, H-4', H-5' and H-4"), 3.65-3.76 (5H, m, H-6, H-6', H-3", H-5", H-6"), 3.80-3.85 (4H, m, H-2', H-3', H-6', H-6"), 3.97 (1H, dd, *J* 1.8 and 3.3 Hz, H-2"), 4.96 (1H, d, *J* 1.6 Hz, H-1"), 5.06 (1H, s, H-1'); $^{13}$C NMR (125 MHz,

methanol-$d_6$) δ 25.51 (C-3), 26.49 and 26.51 (C-4 and NHS $CH_2$)*, 30.05 (C-5), 31.55 (C-2), 63.04 and 63.11 (C-6' and C-6")*, 68.29 (C-6), 68.82 and 69.05 (C-4' and C-4")*, 71.87 (C-2"), 72.18 (C-3'), 72.41 (C-3"), 74.61 (C-5'), 74.96 (C-5"), 80.68 (C-2), 99.93 (C-1'), 104.16 (C-1"), 170.24 (C-1), 171.92 (NHS C=O); $\nu_{max}(cm^{-1})$ = 3362 (broad O-H), 2923 (C-H), 1810 (imide C=O), 1781 (imide C=O), 1731 (C=O); HRMS-ESI $[M+Na]^+$ calculated for $C_{22}H_{35}NO_{15}Na$: 576.1899. Found: 576.1891

**References**

1. Mayer TG, Schmidt RR (1999) Glycosyl phosphatidylinositol (GPI) anchor synthesis based on versatile building blocks - total synthesis of a GPI anchor of yeast. Eur J Org Chem: 1153-1165.
2. Bosone E, Farina P, Guazzi G, Innocenti S, Marotta V, et al. (1983) New synthesis of methyl 7-oxoheptanoate: an useful intermediate for the preparation of 2-(6-methoxycarbonylhexyl)-cyclopent-2-en-1-one. Synthesis-Stuttgart: 942-944.
3. Collot M, Sendid B, Fievez A, Savaux C, Standaert-Vitse A, et al. (2008) Biotin sulfone as a new tool for synthetic oligosaccharide immobilization: application to multiple analysis profiling and surface plasmonic analysis of anti-*Candida albicans* antibody reactivity against alpha and beta (1 -> 2) oligomannosides. J Med Chem 51: 6201-6210.
4. Furneaux RH, Pakulski Z, Tyler PC (2002) New mannotriosides and trimannosides as potential ligands for mannose-specific binding proteins. Can J Chem 80: 964-972.
