# Supplementary material for: Mannosylation of Virus-Like Particles Enhances Internalization by Antigen Presenting Cells
Source: PLoS One. 2014 Aug 14;9(8):e104523. doi: 10.1371/journal.pone.0104523 (PMC4133192; doi:10.1371/journal.pone.0104523)

Data S2 (A). Compound **3** 500 MHz ^{1}H NMR $CDCl_3$

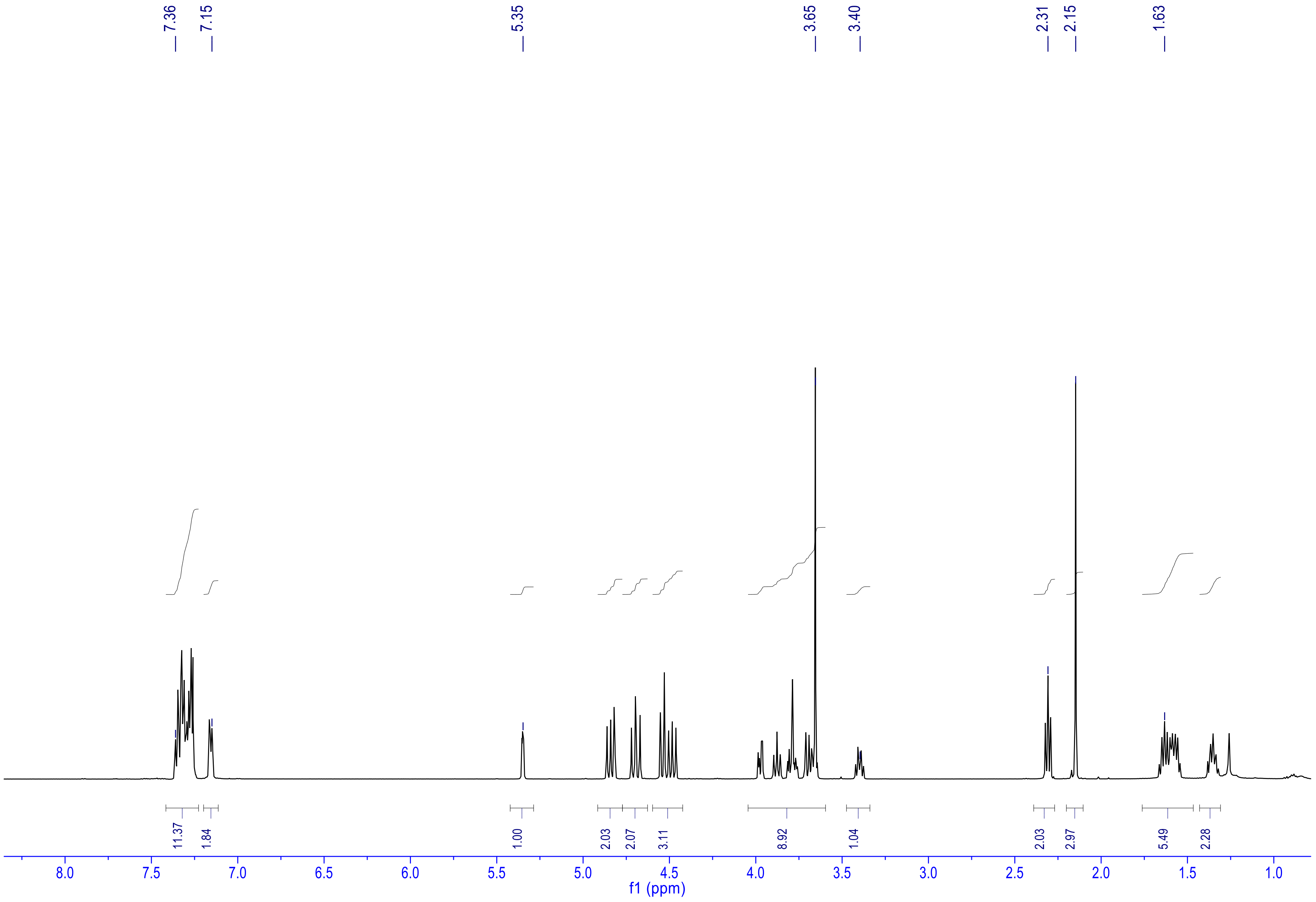

Data S2 (B). Compound **3** 125 MHz ^{13}C NMR $CDCl_3$

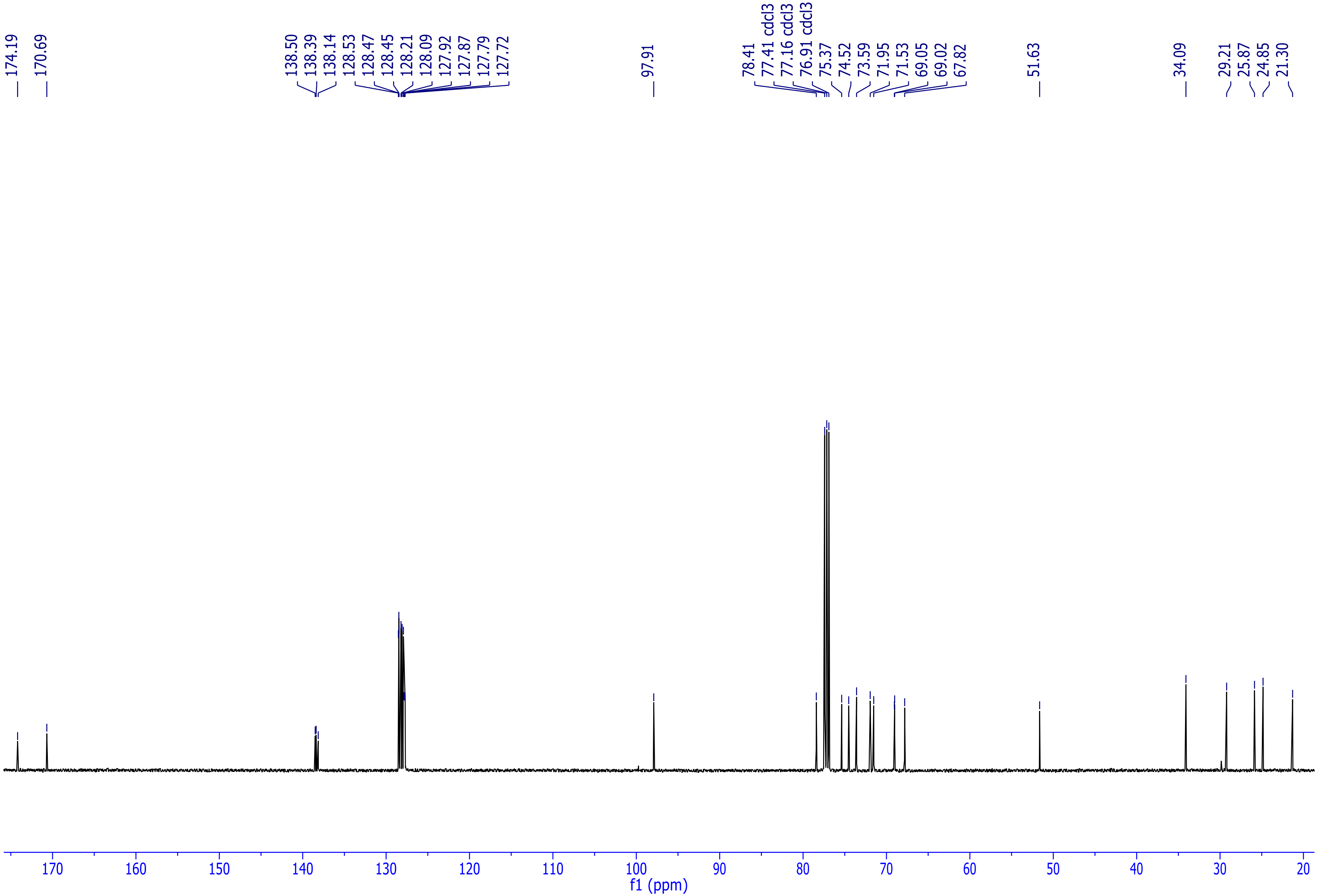

Data S2 (C). Compound **4** 500 MHz ^{1}H NMR $CDCl_3$

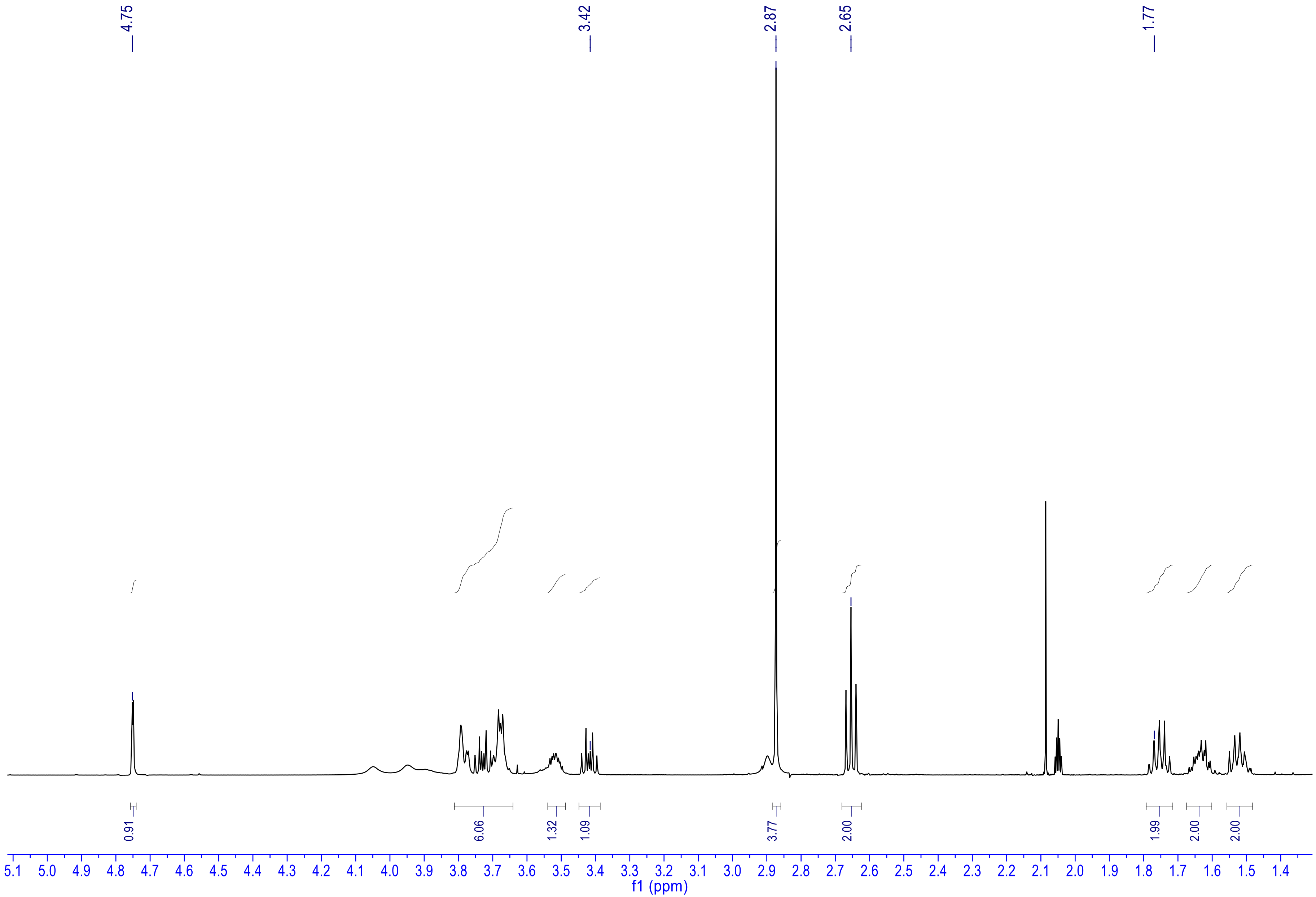

Data S2 (D). Compound **4** 125 MHz ^{13}C NMR $CDCl_3$

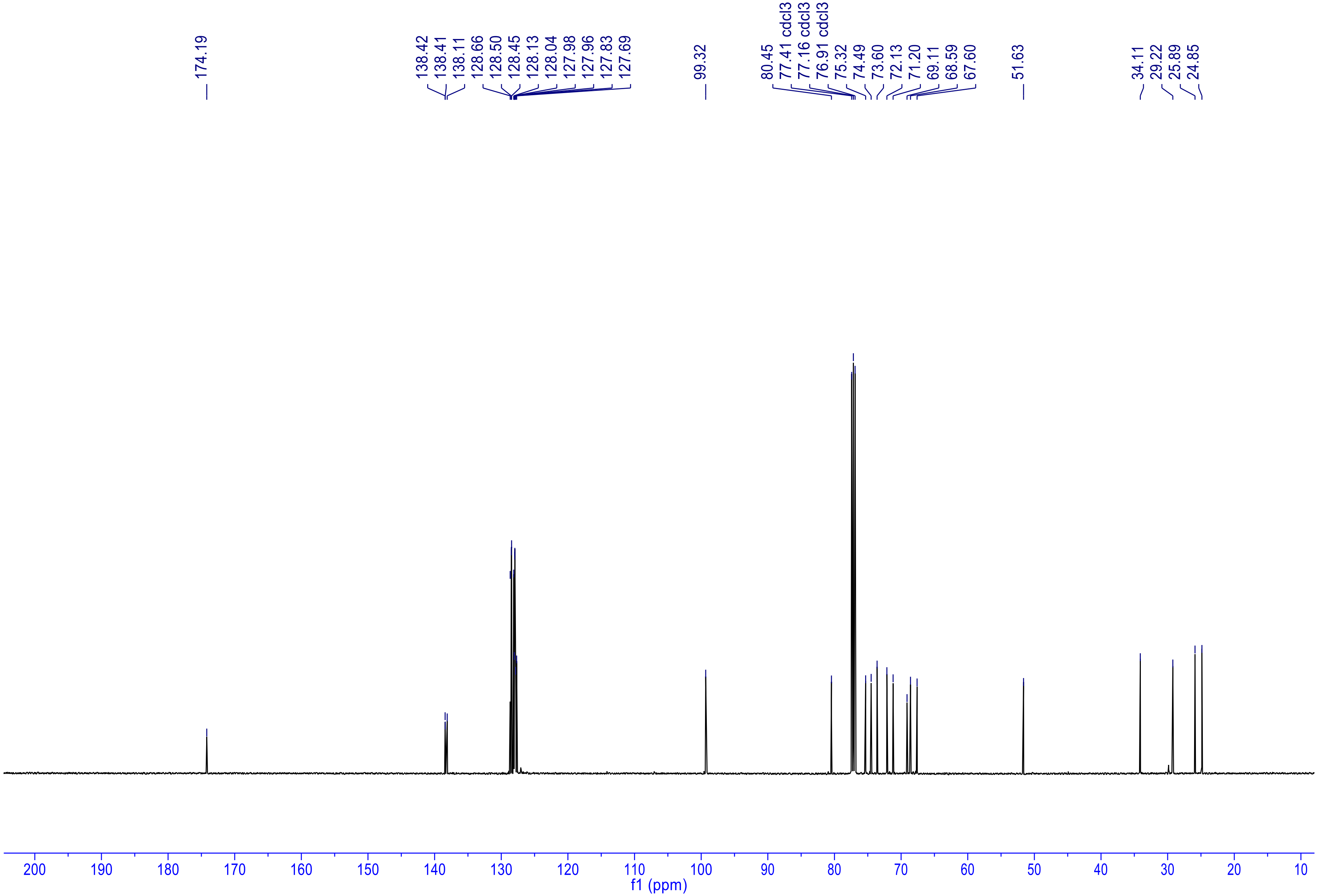

Data S2 (E). Compound **5** 500 MHz ^{1}H NMR $CDCl_3$

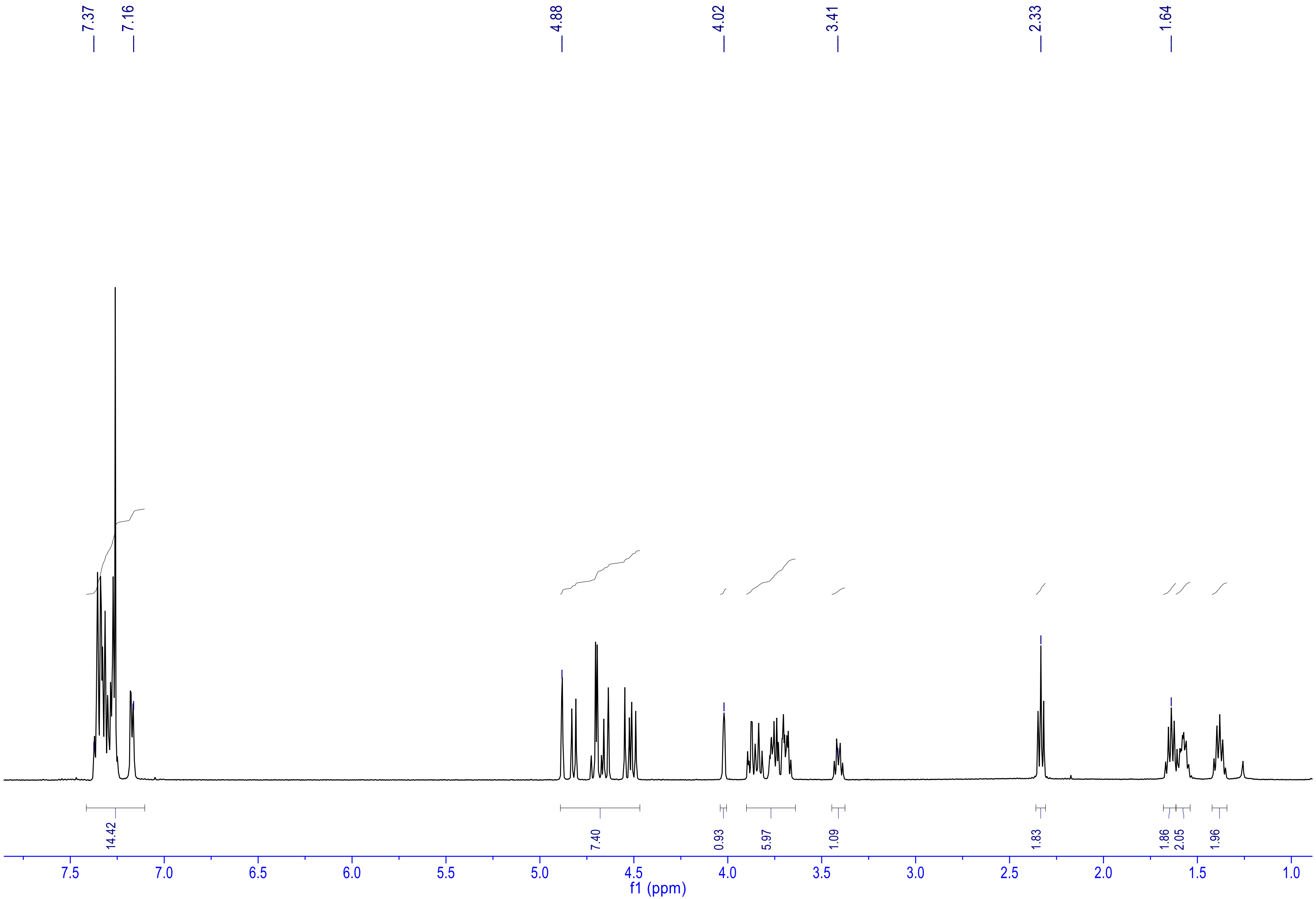

Data S2 (F). Compound **5** 125 MHz ^{13}C NMR $CDCl_3$

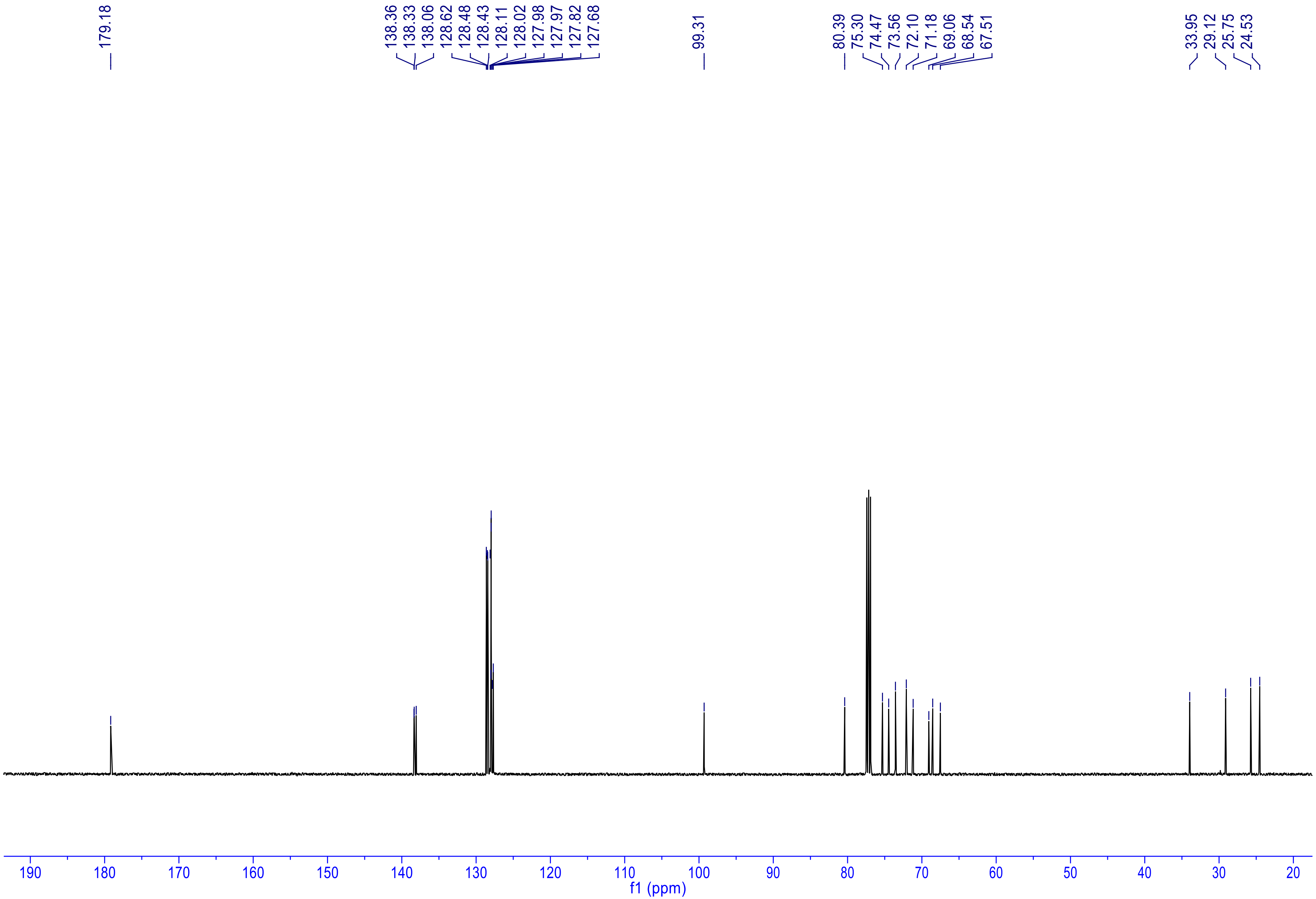

Data S2 (G). Compound **6** 500 MHz ^{1}H NMR $CDCl_3$

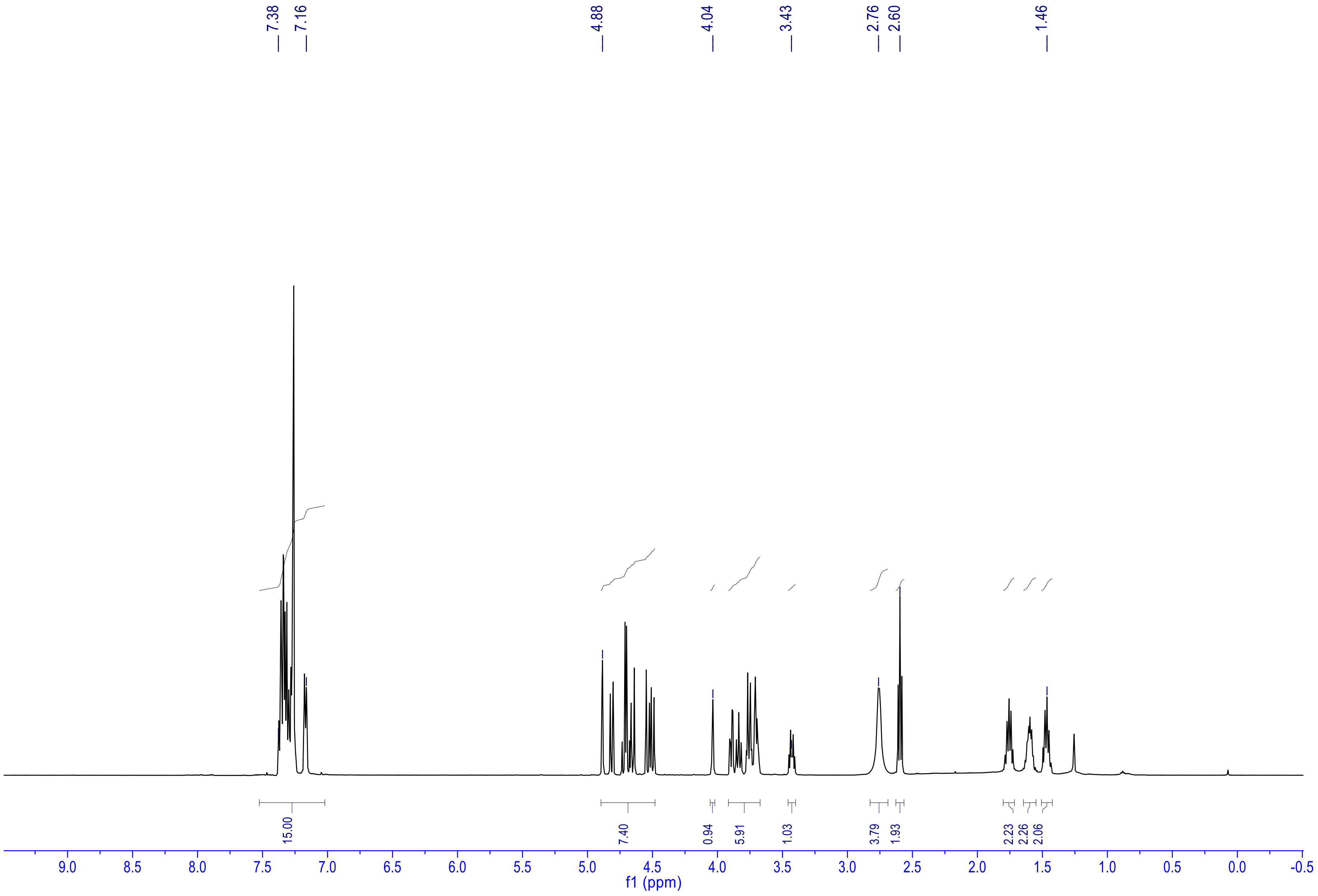

Data S2 (H). Compound **6** 125 MHz ^{13}C NMR $CDCl_3$

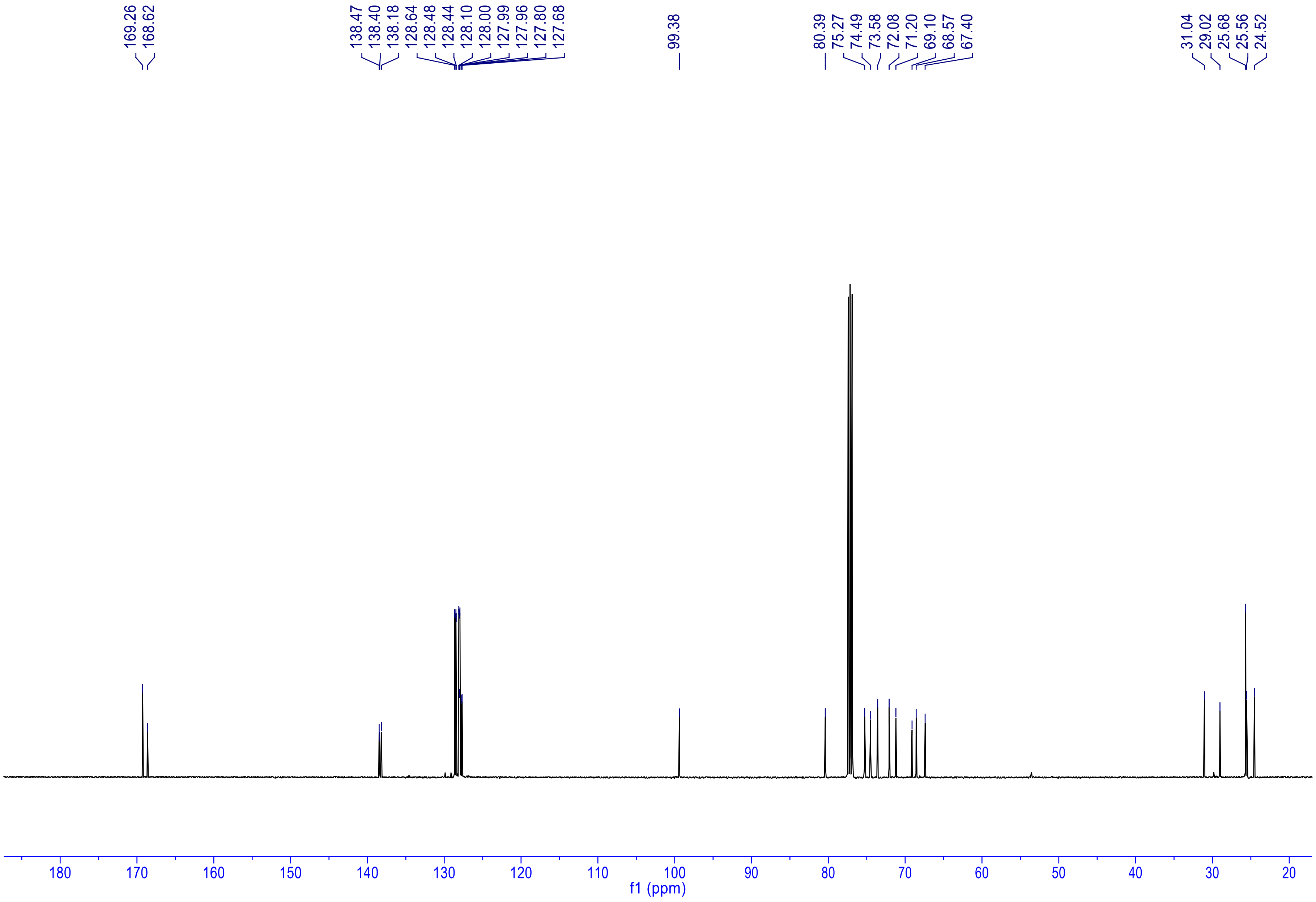

Data S2 (I). Compound **7** 500 MHz ^{1}H NMR $CDCl_3$

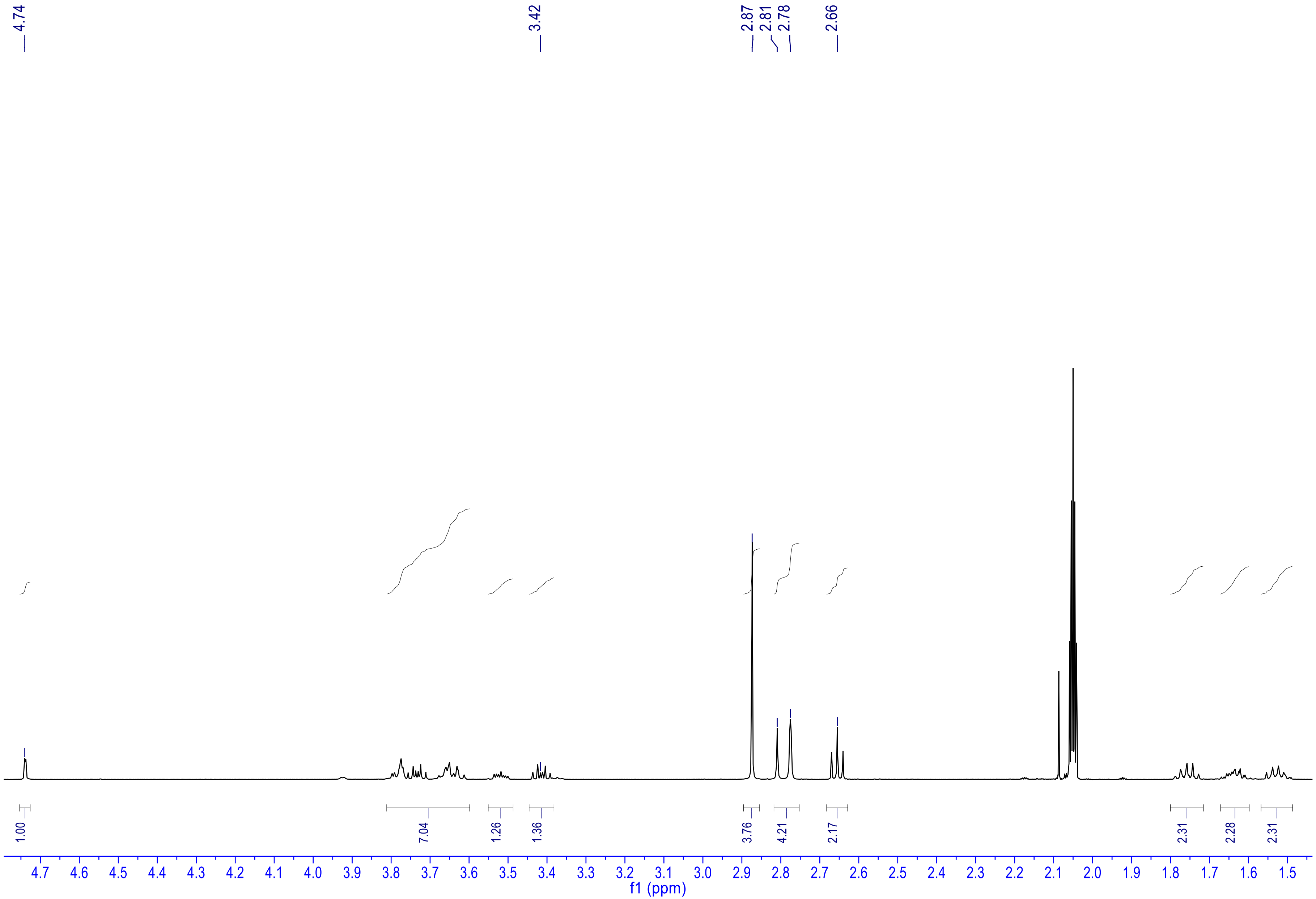

Data S2 (J). Compound **7** 125 MHz ^{13}C NMR $CDCl_3$

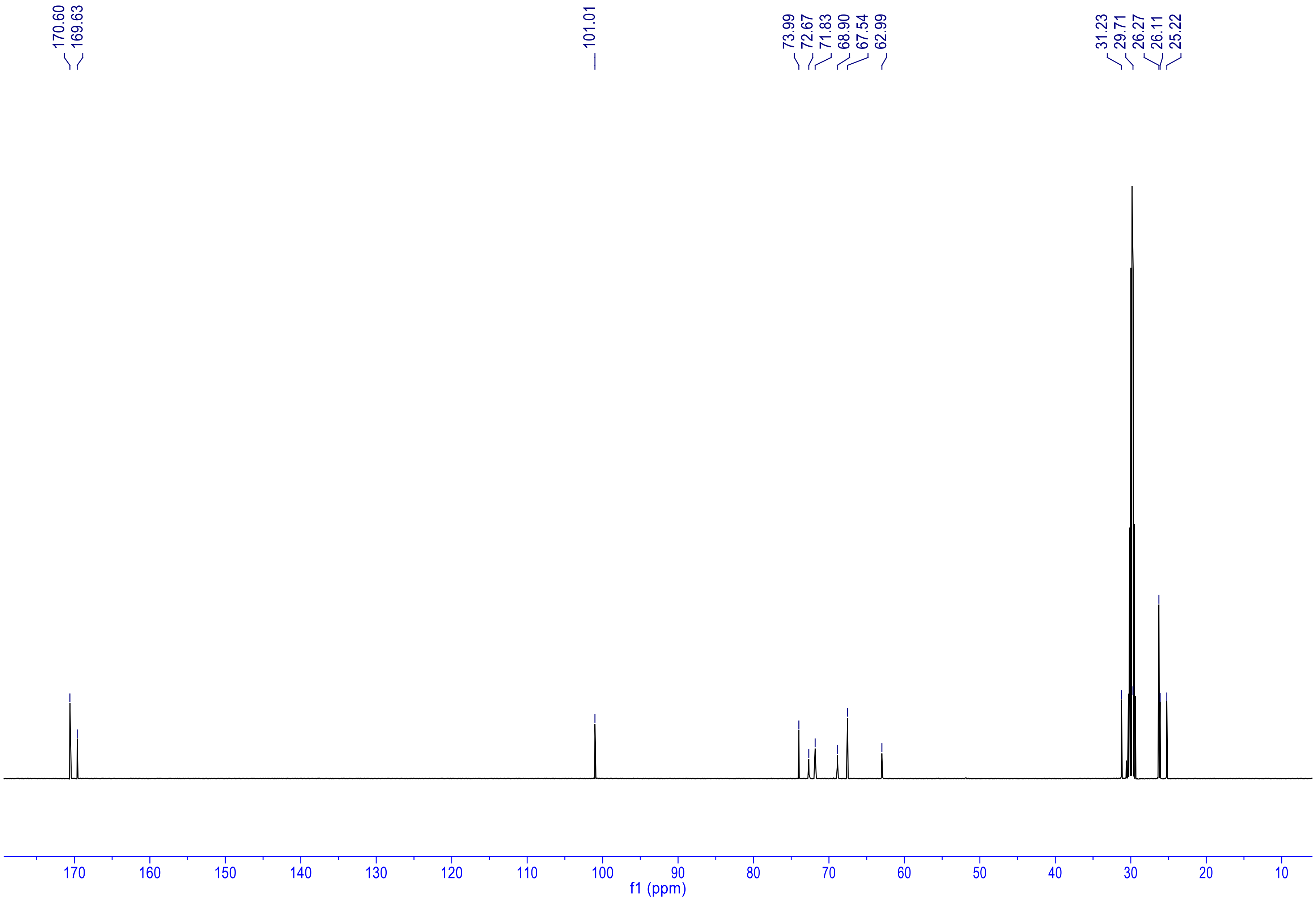

Data S2 (K). Compound **8** 500 MHz ^{1}H NMR $CDCl_3$

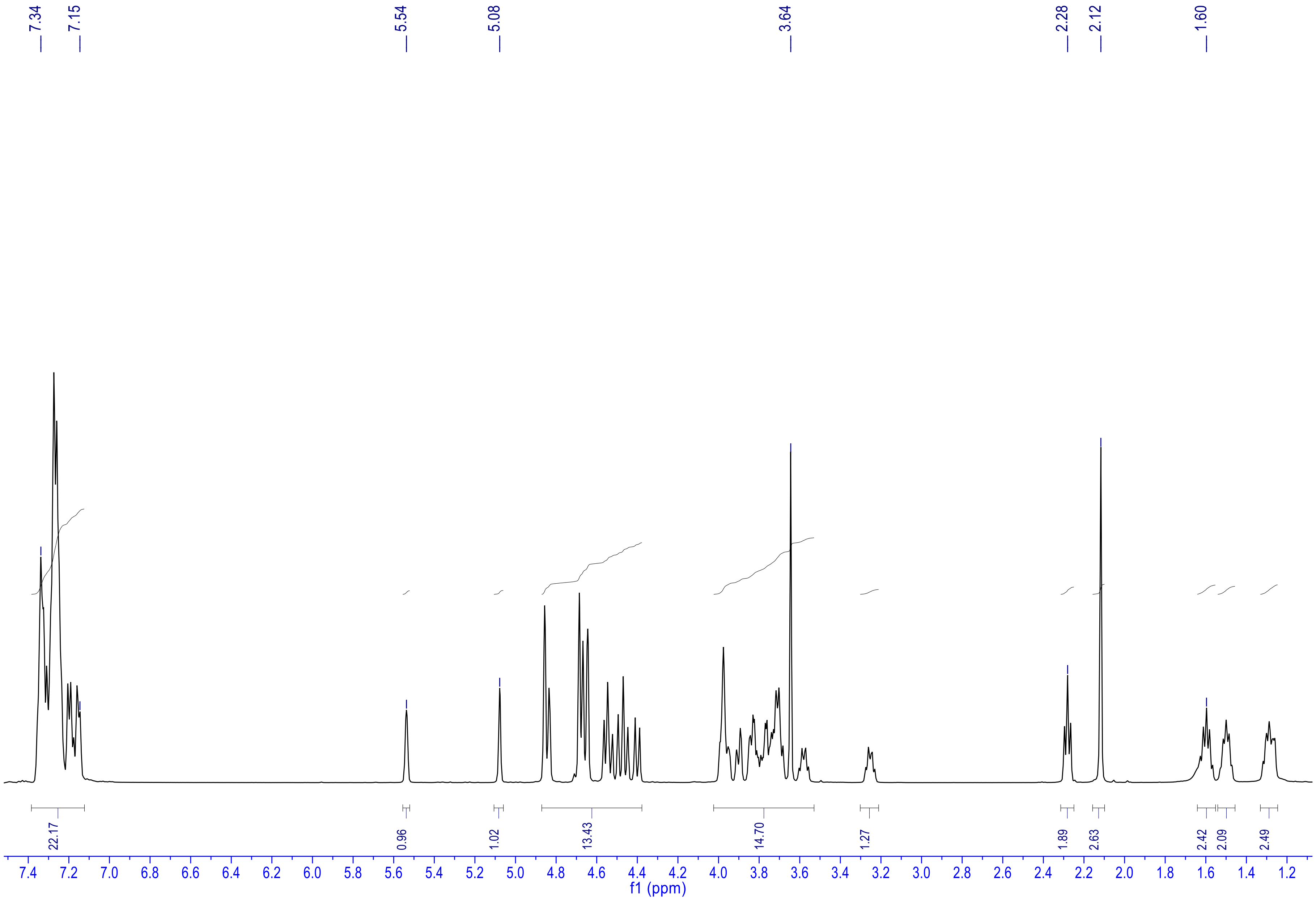

Data S2 (L). Compound **8** 125 MHz ^{13}C NMR $CDCl_3$

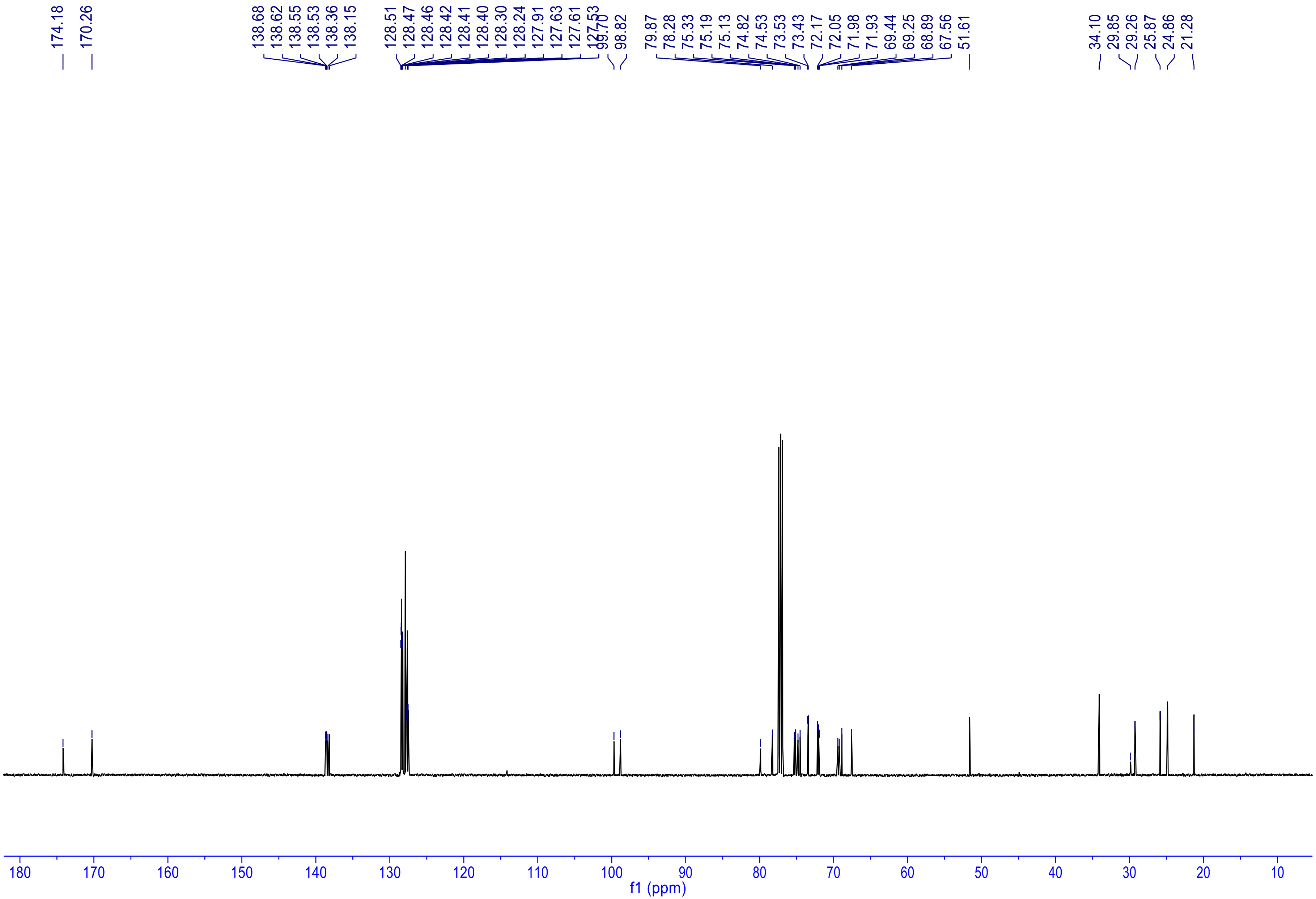

Data S2 (M). Compound **9** 500 MHz ^{1}H NMR $CDCl_3$

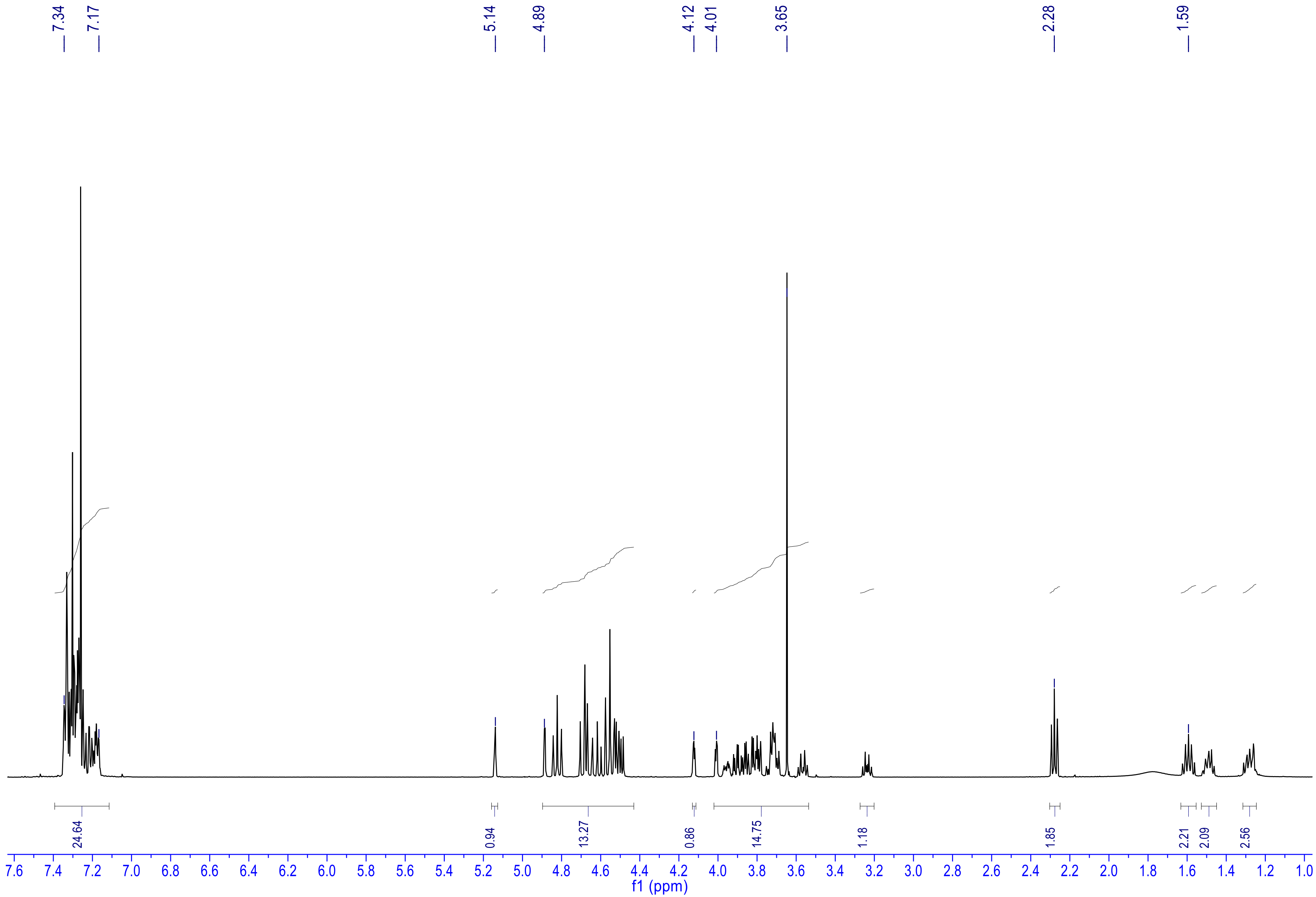

Data S2 (N). Compound **9** 125 MHz ^{13}C NMR $CDCl_3$

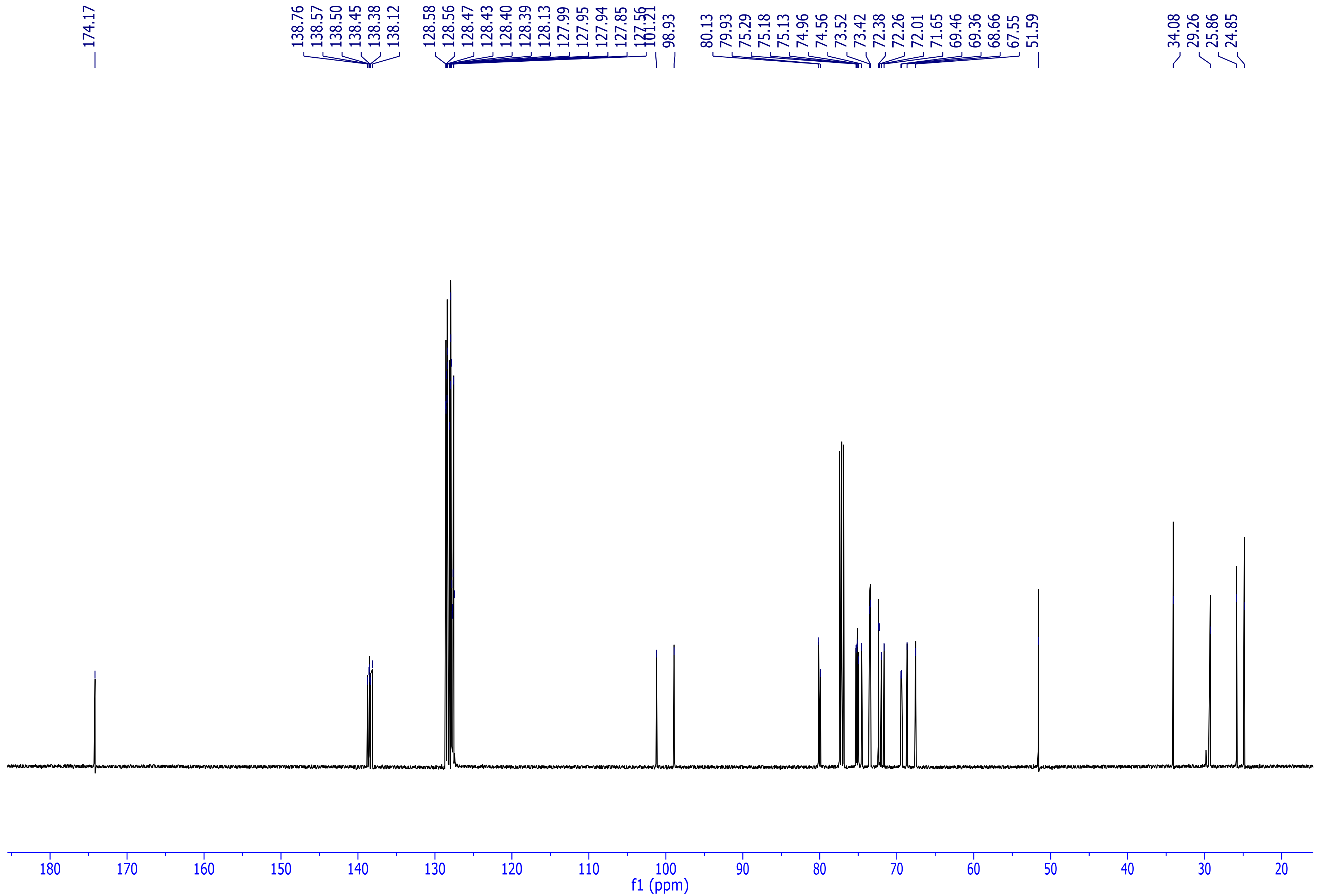

Data S2 (O). Compound **10** 500 MHz ^{1}H NMR $CDCl_3$

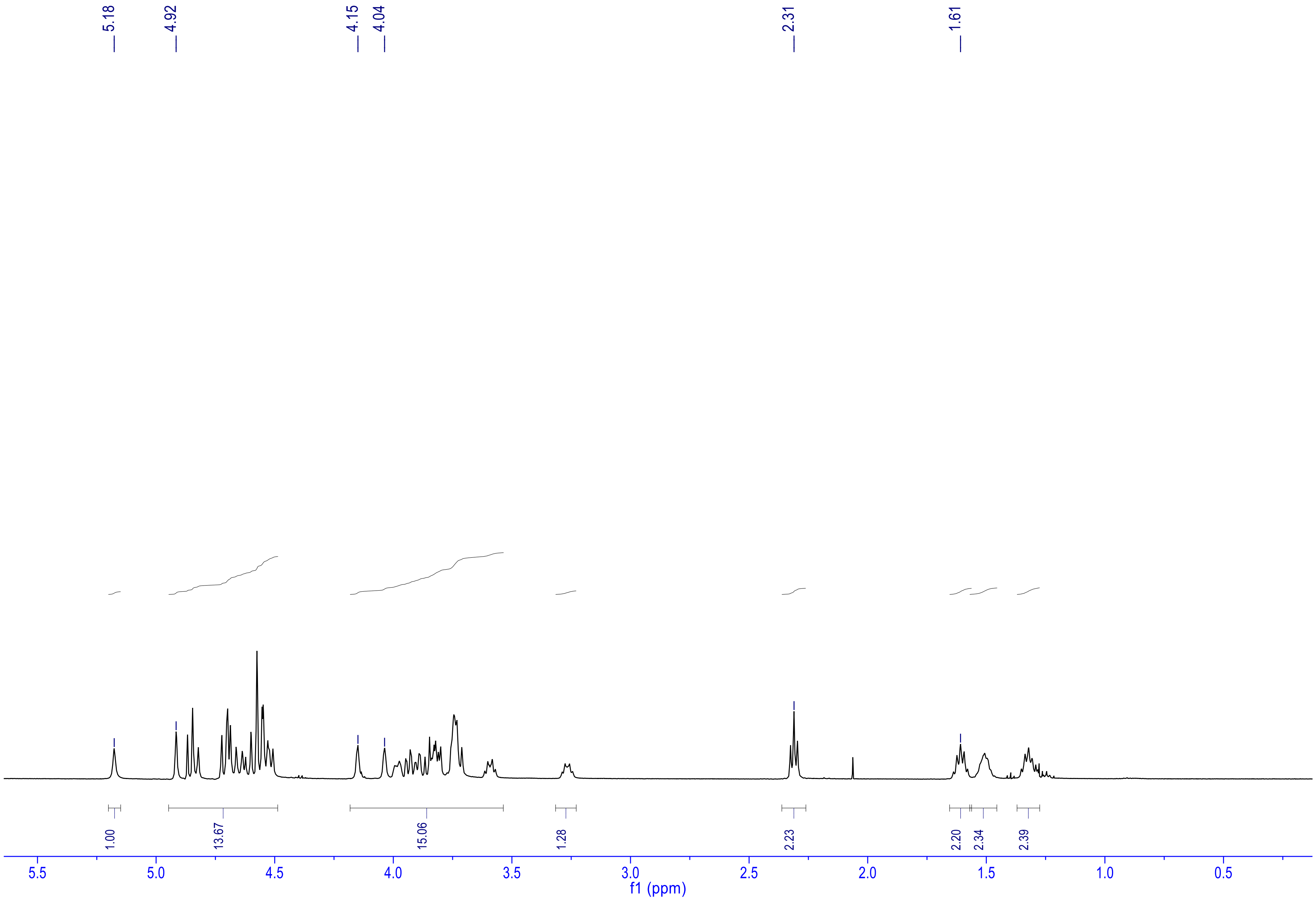

Data S2 (P). Compound **10** 125 MHz ^{13}C NMR $CDCl_3$

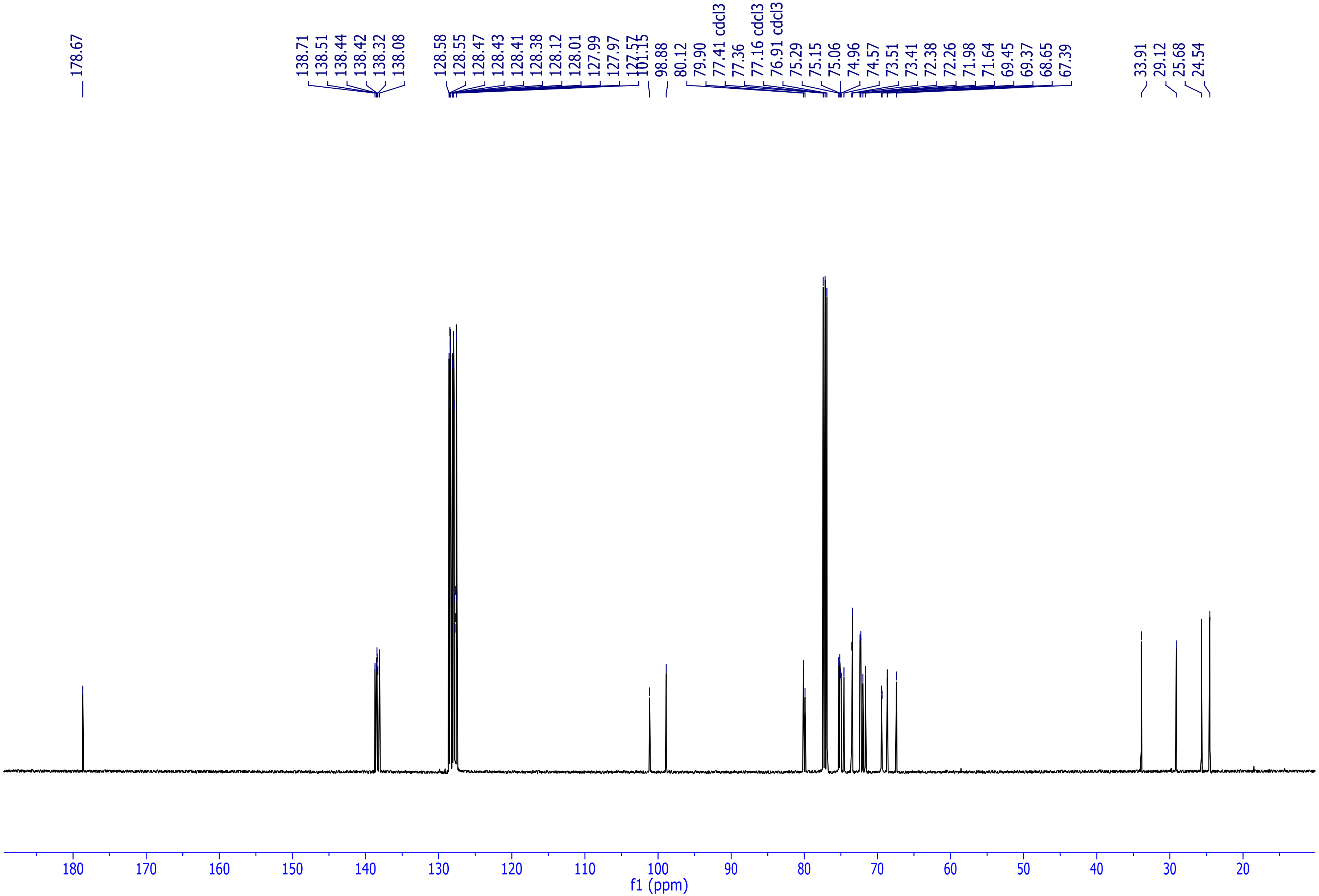

Data S2 (Q). Compound **11** 500 MHz ^{1}H NMR $CDCl_3$

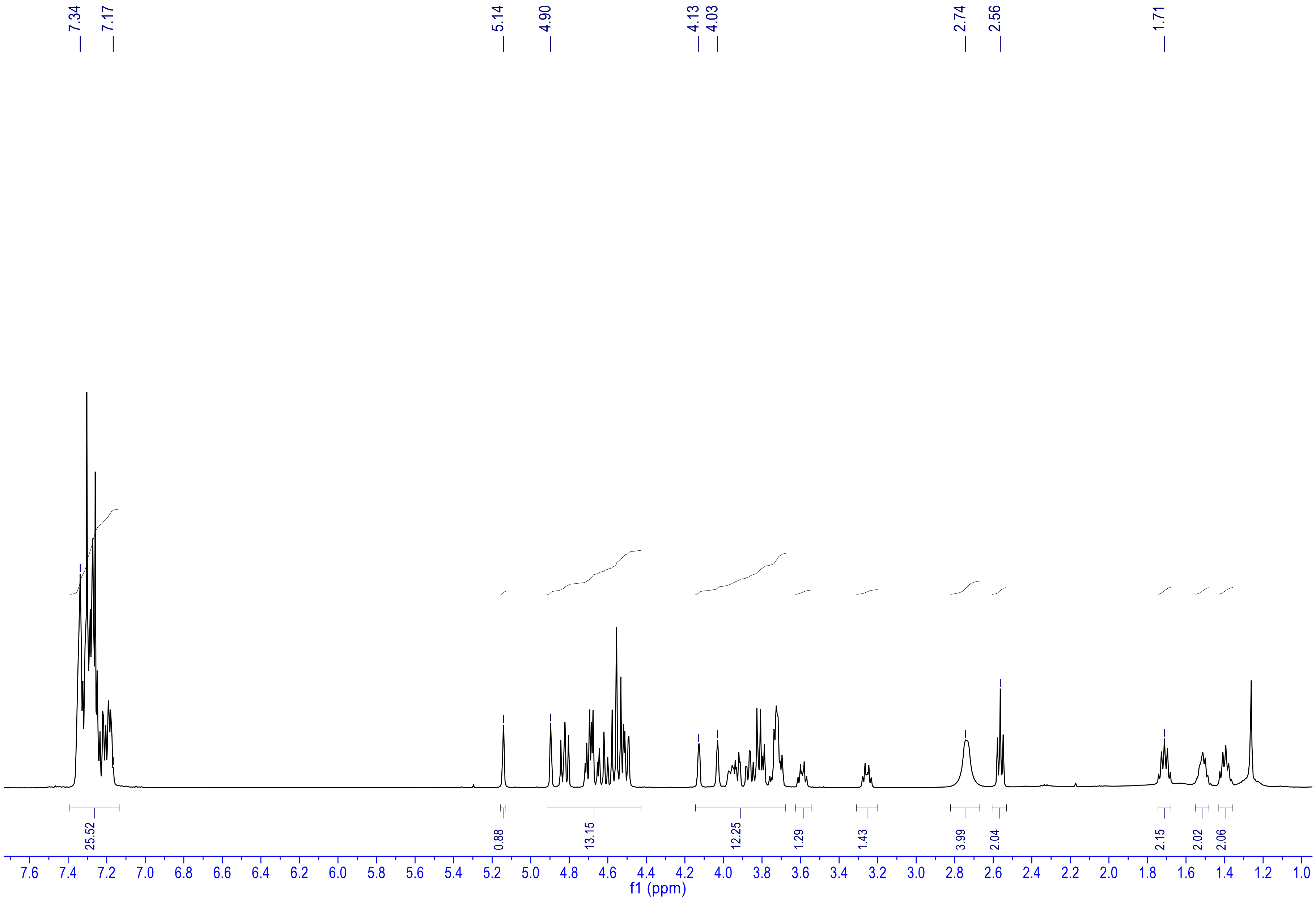

Data S2 (R). Compound **11** 125 MHz ^{13}C NMR $CDCl_3$

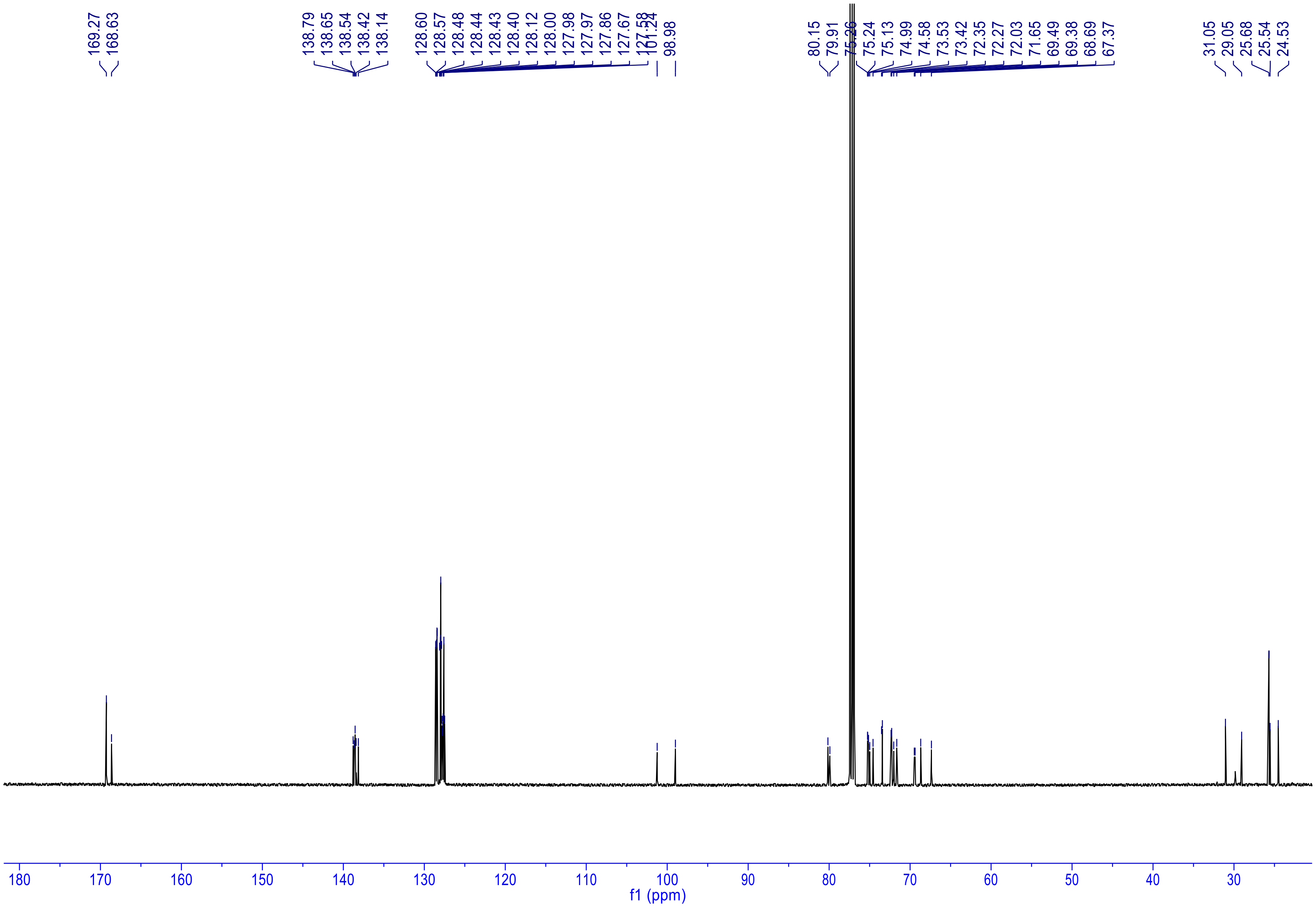

Data S2 (S). Compound **12** 500 MHz ^{1}H NMR $CDCl_3$

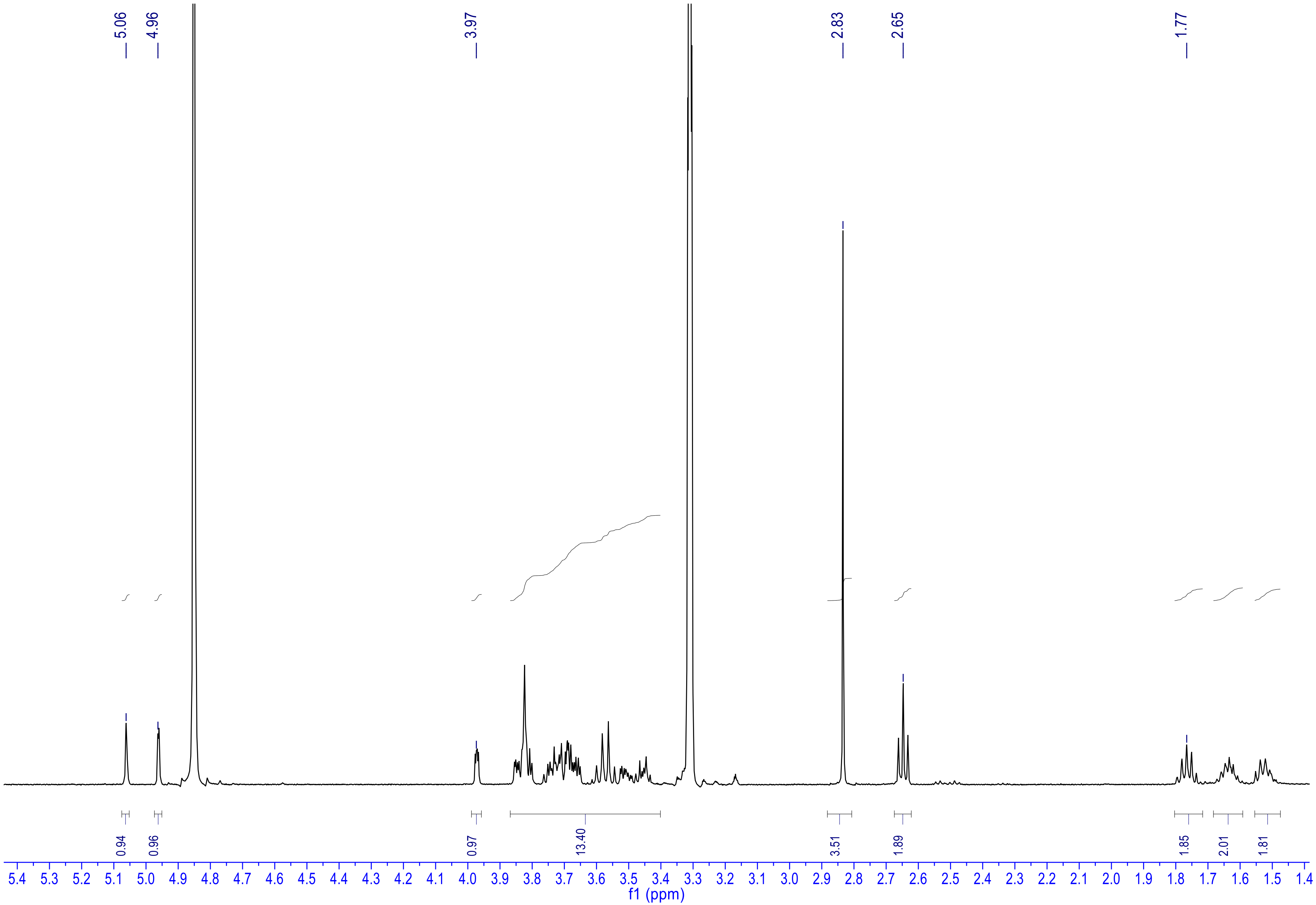

Data S2 (T). Compound **12** 125 MHz ^{13}C NMR $CDCl_3$

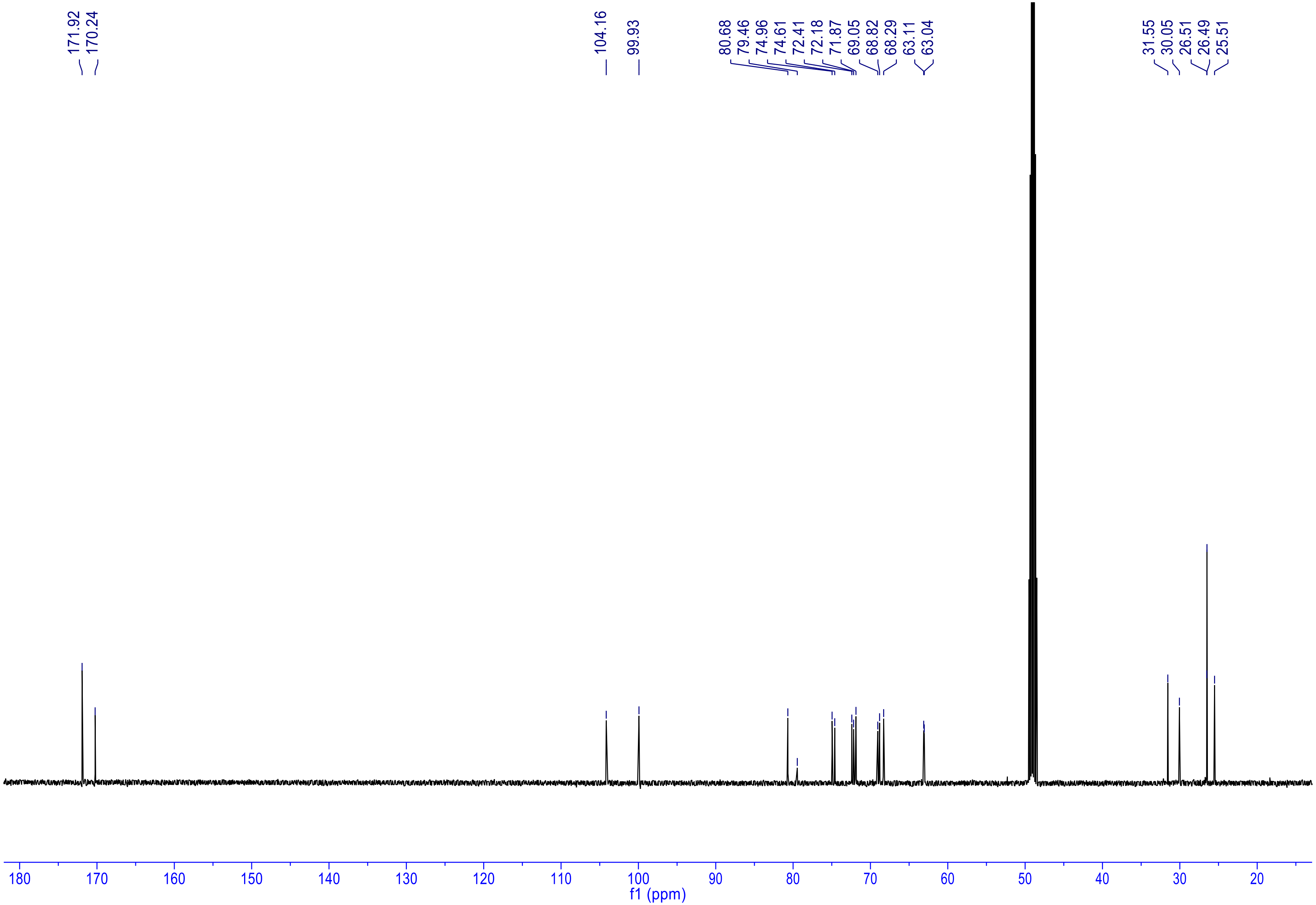

Supplement: Data S2 — 1H and 13C NMR spectra of compounds 3–12. (PDF) [file pone.0104523.s003.pdf]
